# Surveying lncRNA-lncRNA cooperations reveals dominant effect on tumor immunity cross cancers

**DOI:** 10.1038/s42003-022-04249-0

**Published:** 2022-12-03

**Authors:** Tingting Shao, Yunjin Xie, Jingyi Shi, Changbo Yang, Haozhe Zou, Yongsheng Li, Juan Xu, Xia Li

**Affiliations:** 1 College of Bioinformatics Science and Technology, Harbin Medical University, Harbin, 150081 China; 2 Key Laboratory of Tropical Translational Medicine of Ministry of Education, College of Biomedical Information and Engineering, Hainan Women and Children’s Medical Center, Hainan Medical University, Haikou, 571199 China

**Keywords:** Computational biology and bioinformatics, Tumour immunology

## Abstract

Long non-coding RNAs (lncRNAs) can crosstalk with each other by post-transcriptionally co-regulating genes involved in the same or similar functions; however, the regulatory principles and biological insights in tumor-immune are still unclear. Here, we show a multiple-step model to identify lncRNA-lncRNA immune cooperation based on co-regulating functional modules by integrating multi-omics data across 20 cancer types. Moreover, lncRNA immune cooperative networks (LICNs) are constructed, which are likely to modulate tumor-immune microenvironment by regulating immune-related functions. We highlight conserved and rewired network hubs which can regulate interactions between immune cells and tumor cells by targeting ligands and activating or inhibitory receptors such as *PDCD1*, *CTLA4* and *CD86*. Immune cooperative lncRNAs (IC-lncRNAs) playing central roles in many cancers also tend to target known anticancer drug targets. In addition, these IC-lncRNAs tend to be highly expressed in immune cell populations and are significantly correlated with immune cell infiltration. The similar immune mechanisms cross cancers are revealed by the LICNs. Finally, we identify two subtypes of skin cutaneous melanoma with different immune context and prognosis based on IC-lncRNAs. In summary, this study contributes to a comprehensive understanding of the cooperative behaviours of lncRNAs and accelerating discovery of lncRNA-based biomarkers in cancer.

## Abstract (web summary)

LncRNA-lncRNA immune cooperative regulations are identified by integrating multi-omics data across 20 cancer types and the roles of immune cooperative lncRNAs in tumor-immune functions are explored.

## Introduction

Long non-coding RNAs (lncRNAs) are defined as transcripts of more than 200 nucleotides that do not encode proteins, which play crucial roles in diverse biological processes, particularly in diseases such as cancer^1–3^. The function and biological relevance of the lncRNAs in cancer remain enigmatic. Recent studies have suggested that lncRNAs play crucial roles not only in the occurrence and development of cancer with tumor-suppressive and oncogenic activities, but also in cancer immunity, including immune activation and immune cells infiltrating into cancer tissues^4^. For example, *BHLHE40-AS1* has been found to support early breast cancer progression by creating an immune-permissive microenvironment^5^. These immune-related lncRNAs could change the tumor-immune microenvironment by regulating target genes in cancer, including immune cell infiltration. ImmLnc has been introduced for identifying immune-related lncRNAs and multiple lncRNAs tend to co-regulate the same immune pathways in cancer^6^. Immune checkpoint-associated lncRNAs that were involved in key immune response and immune cell receptor signaling pathways were identified based on coding genes correlated with lncRNA expression in breast cancer^7^. Together, these studies suggest that lncRNAs may cooperatively regulate the same immune pathways and participate in cancer immunity.

In our previous studies, we have found extensive synergistic regulation among miRNAs in multiple cancer types, similarly, lncRNAs could crosstalk with each other by co-regulating genes involved in the same or similar functions^8^. Increasing studies have revealed cooperative regulation among lncRNAs in cancer. Dozens of lncRNAs, including *OIP5-AS1*, *TUG1*, *NEAT1*, *MALAT1*, *XIST*, and *TSIX*, have been inferred to synergistically regulate cancer genes and pathways in multiple tumors^9^. Wu et al. found that *HULC* cooperates with *MALAT1* to aggravate liver cancer stem cells proliferation and growth^10^. The lincRNA genomic clusters were systematically identified in pan-cancer and found to be co-expressed and synergistically involved in pathological processes^11^. In the functional analysis of lncRNAs, groups of lncRNAs have been identified that are associated with specific cellular processes, suggesting that lncRNA clusters may co-regulate biological processes by cooperatively regulating genes^12–16^. These observations indicate the presence of cooperative lncRNA regulation, and investigation of the potential functional effects of lncRNA cooperative regulation is interesting. However, it is a challenge to identify lncRNA co-regulation based on experimental methods because of the large number of lncRNA combinations. Context-specific lncRNA co-regulation will provide a better approach for inferring the function of lncRNAs in cancer. It is worth to further exploring the functions, regulatory roles, and biological insights of the lncRNA-lncRNA co-regulation in cancer.

To systematically explore the crosstalk among lncRNAs, cancer-context lncRNA-lncRNA cooperative regulations were identified by integrating multi-omics data based on co-regulating functional modules. We summarized the principles of lncRNA cooperative regulation in immune-related functions. LncRNA immune cooperative networks (LICNs) were further constructed. We explored the roles of hub immune cooperative lncRNAs (IC-lncRNAs) in tumorigenesis and the interaction between immune cells and tumor cells. We subsequently characterized IC-lncRNAs from expression in immune cell populations and immune cell infiltration. The cancer clusters with similar immune mechanisms were revealed based on the structure of the LICNs and expression of IC-lncRNAs. Finally, new immune subtypes of Skin Cutaneous Melanoma (SKCM) were identified based on six IC-lncRNAs (*RP11-71G12*, *RP11-555F9*, *RP11-367G6*, *ITGB2-AS1*, *AP000233,* and *AL928768*). These analyses and validations of lncRNA-lncRNA cooperative regulation contribute to a comprehensive understanding of the cooperative behaviors of lncRNAs in tumor-immune microenvironments.

## Results

### LncRNAs dominantly co-regulate immune-related functions across cancers

**Fig. 1:**
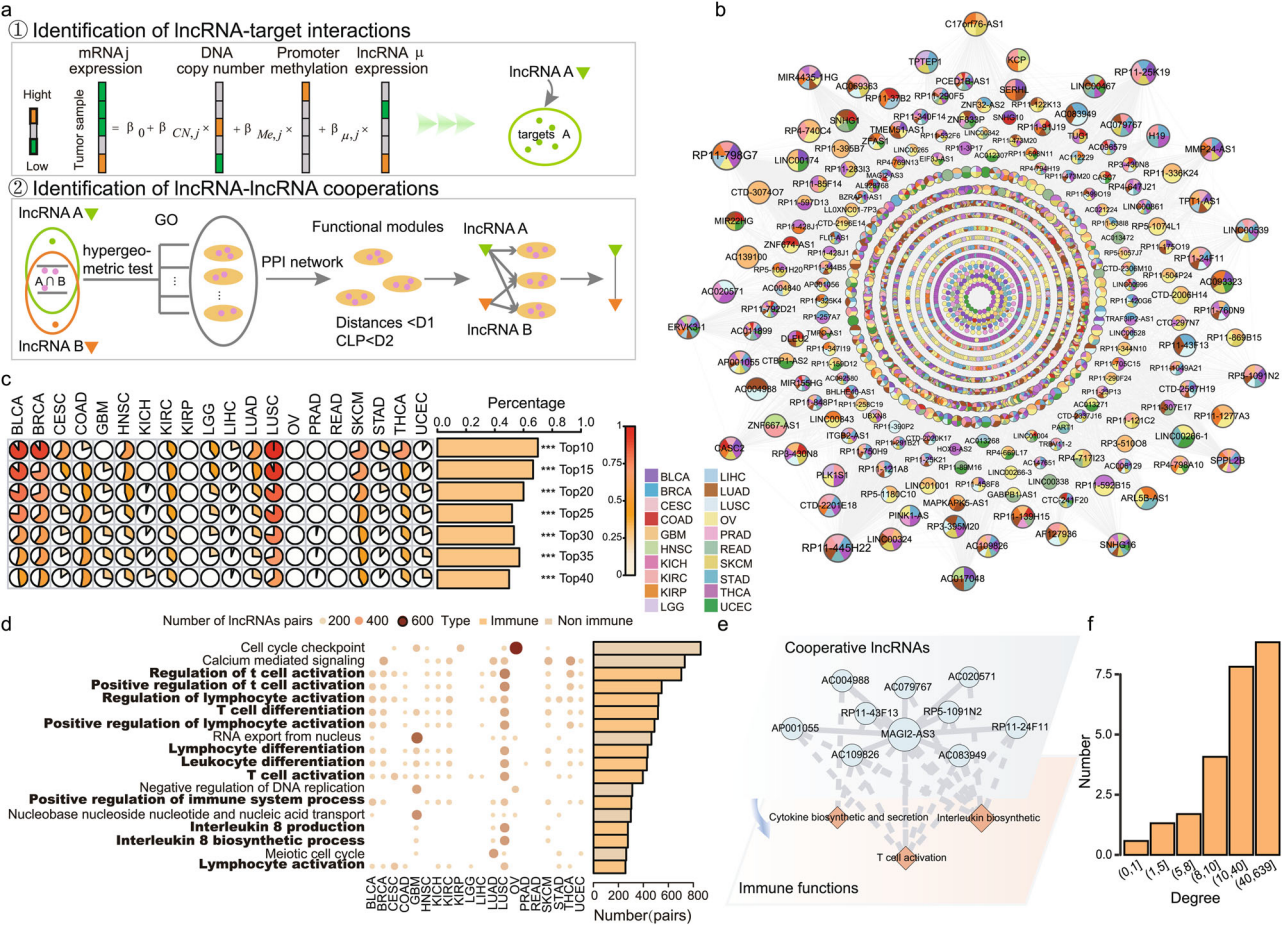
LncRNAs dominantly co-regulate immune-related functions across cancers. **a** A work flow for identifying lncRNA-lncRNA co-regulations. **b** The lncRNA-lncRNA cooperative network in pan-cancer. A node indicates a lncRNA, a color pie chart shows cancers in which the lncRNA occurred and size of the node shows the number of connected nodes. An edge indicates a cooperative regulation between lncRNAs. **c** The percentage of immune functions at the top of all the functions in each cancer type and pan-cancer. Two-sided fisher’s exact test was used to analyze whether or not immune functions are near the top. ***p < 0.001. The number of samples were displayed in Supplementary Table 1. **d** The bubble plot shows the number of lncRNA cooperative pairs co-regulating each function across cancer types. The bar plot shows the total number of lncRNA pairs co-regulating each function in pan-cancer network. The functions in the black font and the dark yellow color are immune-related functions. **e** An example of a sub-network consisting of cooperative lncRNAs and co-regulated immune functions in LUSC. **f** The number of immune functions co-regulated by lncRNAs connected by a different number of neighbors in the pan-cancer.

To explore the function of lncRNAs, we proposed a multiple-step model to identify lncRNA-lncRNA functional cooperation in each cancer type (Supplementary Fig. 1 and Fig. 1a). We first identified the cancer-context-specific lncRNA-target pairs by integrating multi-omics data. The lncRNA-lncRNA cooperations were further identified based on the cancer-context lncRNA-target regulation across 20 cancer types (Supplementary Table 1)^17^. In each cancer type, one lncRNA pair was considered as cooperation, if and only if they could co-regulate at least a functional module, which consisted of their shared targets. We identified 6449 lncRNA-lncRNA cooperation pairs among 2145 lncRNAs. The number of cooperative lncRNAs accounts for only 16.86% of all the lncRNAs, but these lncRNAs are tightly connected and assembled into a lncRNA-lncRNA cooperative network in pan-cancer (Fig. 1b). Then, we found that ~45.13% (968/2145) of lncRNAs exhibit cooperation in at least two cancers, and 110 lncRNAs participate in co-regulation in more than six cancers (Supplementary Data 1). On the other hand, 94.73% (6109/6449) of lncRNA cooperative interactions are cancer-specific (Supplementary Fig. 2). These results indicate that although nearly half of lncRNAs play cooperative roles in more than one cancer, the cooperative partners of these lncRNAs might be changed in different cancers. Furthermore, by revealing the topological structure with a power-law degree distribution and higher clustering coefficients than randomly linked networks (Supplementary Fig. 3a, b), the cooperative network of pan-cancer exhibits the scale-free and modular characteristics, indicating that lncRNA-lncRNA cooperative interactions influence each other and effectively exchange regulated information both at a global and at a local scale. Through analysis of the genomic location and expression, we found that the majority of the cooperative pairs regulate the same function module in trans (Supplementary Fig. 4, *p* = 0.003) and the cooperative lncRNAs tend to have similar expression patterns in cancer types with similar tissue origin (Supplementary Fig. 5), such as lower-grade glioma (LGG) and glioblastoma multiforme.

To further explore the functional characteristics of cooperative lncRNAs, we ranked the functions co-regulated by lncRNAs in descending order according to the number of cooperative lncRNA pairs. We found that all the top functions are related with cancer development and progression, including cell cycle checkpoint, negative regulation of DNA replication, and especially many immune-related functions. The proportion of immune-related functions increases with the order and is averagely as high as 58% in top 10 to 40 (Fig. 1c). Particularly, the trend of lncRNAs co-regulating immune function is more obvious in lung squamous cell carcinoma (LUSC) and bladder carcinoma (BLCA), and 100% and 90% of top 10 functions are related with immune, respectively. As shown in Fig. 1d, 12 (67%) of the top 18 functions are associated with immune, such as T-cell activation, lymphocyte activation, and interleukin 8 production. Up to 10.7% (696/6449) cooperative lncRNA pairs are involved in regulation of T-cell activation. In LUSC, each of these immune functions is co-regulated by an average of 208 lncRNA pairs. The lncRNA *MAGI2-AS3* could co-regulate cytokine biosynthetic and secretion, interleukin biosynthetic, and T-cell activation with nine lncRNAs, and is overexpressed in LUSC (Fig. 1e). It has been proved that *MAGI2-AS3* could upregulate suppressor cytokine signaling 1 and suppress the proliferation of NSCLC cells^18^. The result suggests that co-regulating immune function with other lncRNAs may be another mechanism in cancer development for *MAGI2-AS3*. Moreover, we found that lncRNAs with more neighbors in the cooperative network tend to co-regulate more immune-related functions (Fig. 1f). These results imply that cooperative lncRNA pairs may contribute to carcinogenesis by regulating immune-related functions in multiple cancers. Our finding may provide a new entry that cooperative lncRNAs have contributions to modify tumor-immune microenvironment.

### LICNs are involved in multiple levels of immune processes

**Fig. 2:**
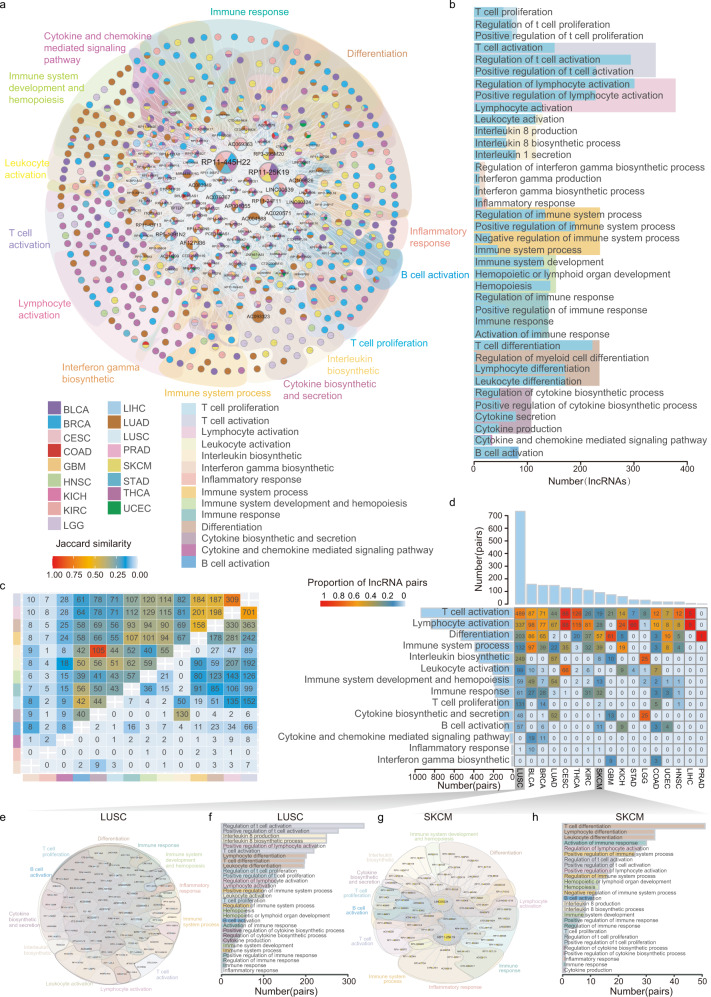
LICNs are involved in multiple levels of immune processes. **a** The LICN in pan-cancer. The shadows in different colors represent immune functions co-regulated by IC-lncRNAs. A node indicates an IC-lncRNA, a color pie chart shows cancers in which the IC-lncRNA occurred and the size of the node shows the number of connected nodes. An edge indicates a cooperative regulation between IC-lncRNAs. **b** The number of IC-lncRNAs co-regulating immune-related functions. These immune functions are summarized into 14 categories. The colorful shadow on the background indicates the 14 immune categories. **c** The upper triangular matrix shows the proportion of IC-lncRNAs shared by any two immune function categories and the integer shows the number of shared IC-lncRNAs. The lower triangular matrix shows the proportion of shared IC-lncRNA co-regulations. **d** The matrix shows the number of IC-lncRNA pairs co-regulating immune functions in each cancer and the color legend indicates the proportion of IC-lncRNA pairs co-regulating each immune function in each cancer. The bar plot shows the number of IC-lncRNA pairs co-regulating immune functions in each cancer or all cancers. **e**, **g** The LINC of LUSC and SKCM respectively. A node indicates an IC-lncRNA and size of the node shows the number of connected nodes. An edge indicates a cooperative regulation between IC-lncRNAs. The shadows in different colors represent immune functions co-regulated by IC-lncRNAs. **f**, **h** The number of immune functions co-regulated by IC-lncRNA cooperative regulations in LUSC and SKCM, respectively.

To further gain insight into the roles of cooperative lncRNAs in immunity, we extracted all the cooperative lncRNA pairs regulating immune-related functions in each cancer (Supplementary Table 1) and constructed LICNs in 17 cancers respectively based on these pairs (Supplementary Fig. 6 and 7). Furthermore, an immune lncRNA-lncRNA cooperative regulatory landscape in pan-cancer was constructed by integrating cancer-specific LICNs (Fig. 2a). This network involves 505 IC-lncRNAs and 1628 IC-lncRNA-IC-lncRNA cooperative interactions across 17 cancer types. Furthermore, by revealing the topological structure with a power-law degree distribution and higher clustering coefficients than randomly linked networks (Supplementary Fig. 8), the LICN of pan-cancer exhibits the scale-free and modular characteristics, indicating that IC-lncRNA cooperative interactions influence each other and effectively exchange regulated information both at a global and at a local scale. Furthermore, we found that the expression of IC-lncRNAs is higher than other lncRNAs in most cancers (Supplementary Fig. 9a). The IC-lncRNAs pairs also exhibit significantly stronger expression correlation than random in 10 cancers (Supplementary Fig. 9b). Thus, we proposed that similar expression patterns might help IC-lncRNAs perform cooperative functions.

Next, we summarized these immune functions into 14 categories and found that these functions are widely distributed, involving multiple levels of immune processes (Fig. 2b). Overall, lymphocyte activation is co-regulated by the largest number of IC-lncRNAs. Moreover, we also found that some function categories with internal connections share more cooperative IC-lncRNAs, such as lymphocyte activation and T-cell activation (Fig. 2c). The trend of the co-regulation for IC-lncRNAs to lymphocyte activation may offer new candidates for cancer immunotherapy. Next, we explored the co-regulation of IC-lncRNAs to immune function categories in each cancer. On average, each cancer type yields 111 co-regulations among 63 lncRNAs participating in the immune process. A higher number of IC-lncRNA co-regulations were identified in the cancer types where immune checkpoint-blocking drugs were applicable in clinical (Fig. 2d)^19^. For example, there are 99 IC-lncRNAs and 734 co-regulation pairs in LUSC (Fig. 2e). These IC-lncRNAs account for ~23.5% (505/2145) of all cooperative lncRNAs (Fig. 2d). In addition, the LICN of SKCM contains 110 cooperations among 73 IC-lncRNAs (Fig. 2g). For example, IC-lncRNA *LINC00324*, which is more highly expressed than others, co-regulates 10 immune-related functions, such as lymphocyte activation, cytokine biosynthetic and secretion, and interleukin biosynthetic with 49 IC-lncRNAs. A previous study concluded that *LINC00324* could regulate the expression of *FasL*, an apoptosis suppressor concentrated in immune cells and cancer cells, which had been shown to play a vital role in immune evasion^20^. Higher number of IC-lncRNAs regulate the T-cell activation and lymphocyte activation across cancer types (Fig. 2d). Particularly, 338 IC-lncRNA pairs are involved in regulation of T-cell activation in LUSC (Fig. 2f). Activation of T cells has become an important way to promote antitumor immune response in lung cancer^21^. Moreover, 51 IC-lncRNAs pairs co-regulate T-cell differentiation in SKCM (Fig. 2h). The dysregulation of T-cell differentiation has been shown in melanoma progression^22^. These results imply that the IC-lncRNAs might play critical roles in the tumor-immune microenvironment.

### Conserved and rewired LICN hubs could regulate tumor-immune cell interactions

**Fig. 3:**
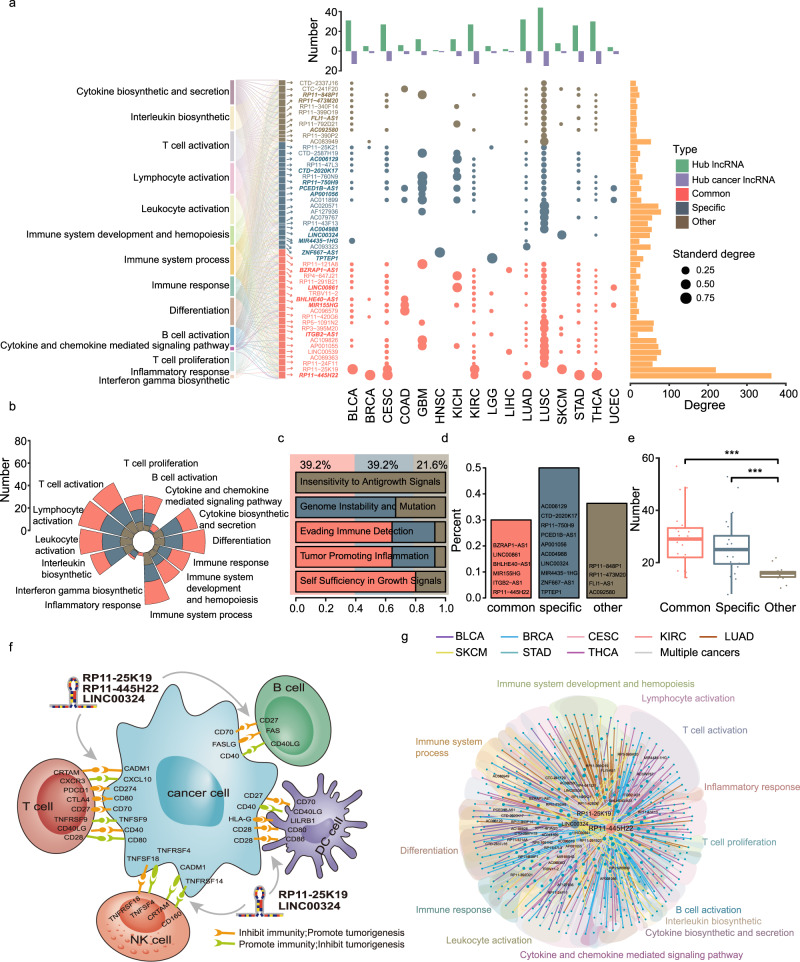
Conserved and rewired LICN hubs could regulate tumor-immune cell interactions. **a** The classification of hub IC-lncRNAs in pan-cancer LICN. The grouped bar plot indicates the number of hub IC-lncRNAs and cancer-related hub IC-lncRNAs in LICNs of cancers. The bar plot in yellow color shows the degree of each hub IC-lncRNA in the pan-cancer LICN. The bubble diagram indicates the degree of each hub IC-lncRNA divided by the total number of IC-lncRNAs in each LICN. The river plot shows the functions co-regulated by each hub IC-lncRNAs. Cancer-related hub IC-lncRNAs are in Italic bold font. **b** The number of hub IC-lncRNAs co-regulating each immune function category in each hub group. **c** The proportion of hub IC-lncRNAs co-regulating each cancer hallmark in each hub group. The colored shadow shows the proportion of three kinds of hub IC-lncRNAs. **d** The proportion of cancer-related hub IC-lncRNAs in each hub category. The cancer-related hub IC-lncRNAs are shown in the plot. **e** The number of drug target genes regulated by different kinds of hub IC-lncRNAs. Two-sided *t* test was used on *n* = 20 common hubs, *n* = 20 specific hubs, *n* = 11 other hubs, ***p < 0.001. The boxplots are shown as median (line), interquartile range (box) and data range or 1.5× interquartile range (whisker), each point indicates a hub IC-lncRNA. **f** A diagram shows the interactions between immune cells and tumor cells modulated by three IC-lncRNAs. The target genes regulated by IC-lncRNAs shown in the plot are receptors and ligands expressed on the surface of immune cells or tumor cells. **g** An immune cooperative regulation sub-network consisting of IC-lncRNA cooperative regulations where the three hub IC-lncRNAs showed in Fig. 3f involved. The shadows in different colors represent immune functions co-regulated by IC-lncRNAs. A node indicates an IC-lncRNA and size of the node shows the number of connected nodes. An edge indicates a cooperative regulation between IC-lncRNAs. Colors of edges indicate cancer types the regulations involved.

A few nodes with a large number of neighbors as hubs hold the nodes together in each network. The presence of hubs seems to be a general feature of all biological networks, and these hubs fundamentally could determine the behavior of networks^23,24^. To investigate the crucial nodes in the pan-cancer LICN, we first identified the top 10% of nodes with the highest connectivity as hub IC-lncRNAs. Then, we assessed the roles of these hub IC-lncRNAs among the different cancer LICNs and grouped these hubs into three categories: common hubs, cancer-specific hubs, and other hubs. The common hubs signify the central roles of IC-lncRNAs in more than one cancer; the specific hubs identify IC-lncRNAs with specific central roles in a given cancer. In total, 39.2% (20/51) of hubs are common hubs, 39.2% (20/51) are specific hubs, and the remaining 21.6% are other hubs. We found that all the hub IC-lncRNAs are extensively involved in immune functions (Fig. 3a). The distribution of three categories of hubs is different across cancers; both LUSC and lung adenocarcinoma (LUAD) contain many common hubs. We further investigated whether three types of hubs have different roles in cancer immune. We found that most of these hubs co-regulate the majority of the immune function categories, such as T-cell activation, cytokine biosynthetic and secretion, and B-cell activation (Fig. 3b). On the other hand, several function categories tend to be specifically co-regulated by different categories of hubs, such as inflammatory response, which is regulated only by the common hubs *AC109826*, *RP11-25K19* and *RP11-420G6*, and interferon-gamma biosynthetic is specifically regulated by the cancer-specific hubs (Fig. 3b). These results suggest that these hub IC-lncRNAs might play important roles in immune-related functions.

We further explored the contribution of the IC-lncRNAs to cancer development. First, we found that 18 out of 51 hub IC-lncRNAs could participate in cancer hallmarks (Fig. 3c). Nine hub IC-lncRNAs, particularly common hubs, regulate evading immune detection. At the same time, these IC-lncRNAs could regulate other non-immunological hallmarks. For example, other hub IC-lncRNA *CTC-241F20* specifically regulates insensitivity to antigrowth signals. The results suggest that IC-lncRNAs may synergistically regulate immune-related functions and other cancer-related processes to involve in the development of cancer. We next found that 20 hub IC-lncRNAs are experimentally validated cancer-related lncRNAs, including 6 common hubs and 10 specific hubs (Fig. 3d, Supplementary Data 2). For example, the common hub *MIR155HG* with 17 partners co-regulating B cell and T-cell activation, cytokine biosynthetic, and secretion has been associated with multiple cancer types, including cervical squamous cell carcinoma and endocervical adenocarcinoma (CESC) and kidney renal clear cell carcinoma (KIRC). *MIR155HG* is also a hub IC-lncRNA respectively in CESC and KIRC LICNs. *MIR155HG* is known as an oncogenic lncRNA and dysregulates by PRDM1 in natural killer/T cell lymphoma^25^. Then, we further investigated the expression of hub lncRNAs. We found that the average expression level of other hubs is higher than common hubs (Supplementary Fig. 10, two-sided *t* test *p* = 0.011). These results suggest that IC-lncRNAs may provide a candidate list of cancer-related lncRNAs. Then, we investigated the association between hub IC-lncRNAs and anticancer drugs. We found that 66 out of 84 Food and Drug Administration-approved anticancer drugs could target hub IC-lncRNA-target genes. The common and specific hubs have significantly more anticancer drug targets than other hubs, respectively with an average of 30 and 26 drug targets (Fig. 3e, two-sided *t* test, *p* = 4.39e-05, *p* = 8.55e-04). For example, some target genes of the common IC-lncRNA *RP11-445H22* have been used as anticancer drugs targets, such as CTLA4 as a target of radotinib in cutaneous melanoma, EGFR and ERBB2 as targets of afatinib in metastatic non-small-cell-lung-cancer. The results suggest that target genes of hub IC-lncRNAs are more likely to be druggable and may be potential targets of anticancer drugs.

Tumor-immune microenvironment is very complex involving a range of cell types and molecular mechanisms, especially tumor-immune cell interactions^26^. We surprisingly found that hub IC-lncRNAs could regulate the genes mediating the direct interaction between tumor cells and immune cells, such as T cells, NK cells, DC cells, and B cells (Fig. 3f and Supplementary Data 3). The common hub *RP11-445H22* has the most immune cooperative partners and could regulate *CRTAM*, *CADM1*, *CXCR3*, *CXCL10*, *PDCD1,* and *CD274* to mediate tumor-T cell interactions as shown in Fig. 3f, which plays a role in promoting tumors and suppressing immunity. Among these receptor genes and ligand genes, *CXCL10* and *CXCR3* receive strong regulation from *RP11-445H22* and the regression coefficient respectively are 0.63 and 0.55 in the above lncRNA-target identification model. *RP11-445H22* is a *KCNK15* and *WISP2* antisense RNA and it has been found as an oncogene in lung cancer and gastric cancer^27,28^. The regulation of tumor-immune cell interactions for *RP11-445H22* may be another mechanism to participate in tumorigenesis. *LINC00324* is a specific hub in SKCM, and could be involved in tumor cell-NK cell interactions by regulating *TNFSF18*, *TNFRSF18*, *CRTAM,* and *CADM1*. Furthermore, these three hubs IC-lncRNAs could co-regulate various immune functions with 363, 220, and 50 partners respectively, such as lymphocyte activation, inflammatory response, and interleukin biosynthetic (Fig. 3g). The regulation of IC-lncRNAs on these genes mediating interactions can be leveraged to understand the crosstalk between tumors and immune cells and promises to develop novel drugs or therapeutic strategies.

### IC-lncRNAs are correlated with immune cell (B cells and T cells) infiltration

**Fig. 4:**
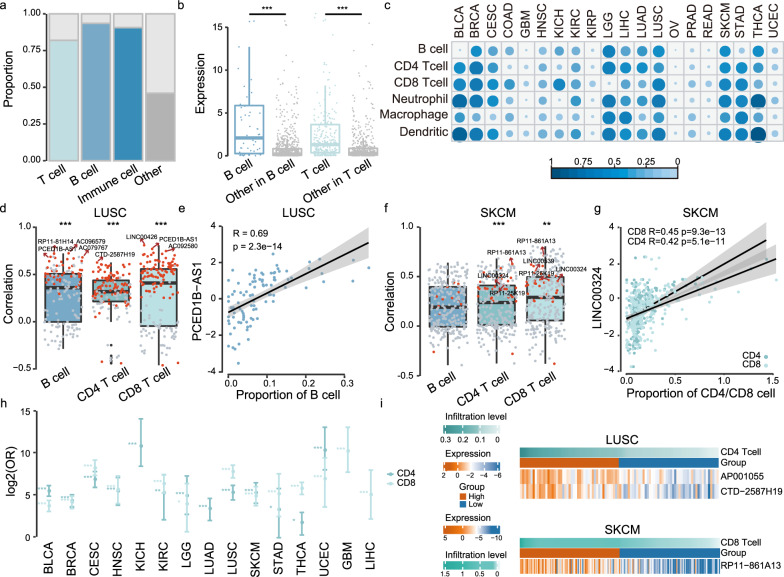
IC-lncRNAs are correlated with immune cell (B cells and T cells) infiltration. **a** The proportion of IC-lncRNAs and other lncRNAs expressed in immune cells and immune cell-related lncRNAs expressed in corresponding immune cells from GEO RNA expression profiles (*n* = 184 samples). The genes in which expression was zero in >80% of samples were abandoned. Two-sided fisher’s exact test was used to analyze whether or not the IC-lncRNAs tend to be expressed in immune cells. **b** The differences of expression between the IC-lncRNAs (*n* = 58 B cell-related lncRNAs, *n* = 290 T cell-related lncRNAs) in corresponding immune cells and other lncRNAs in immune cells using the GEO data were calculated by two-sided Wilcoxon-Mann-Whitney test. ***p < 0.001. The boxplots are shown as median (line), interquartile range (box), and data range or 1.5× interquartile range (whisker), each point indicates a lncRNA. **c** The proportion of cooperative lncRNAs of which expression is significantly related to immune cell infiltration in each cancer. The Spearman’s rank correlation coefficients were calculated between the expression of cooperative lncRNAs and the immune cell infiltration and cutoffs |R| >0.3 and *p* < 0.05. **d**, **f** The Spearman’s rank correlation coefficients between the expression of cooperative lncRNAs (*n* = 151 in LUSC, *n* = 267 in SKCM) and immune cell infiltration in LUSC and SKCM respectively. Each red dot represents correlation coefficient of an IC-lncRNA co-regulating corresponding immune cell-related functions. Two-sided Wilcoxon-Mann-Whitney test was used. ***p < 0.001, **p < 0.01. The boxplots are shown as median (line), interquartile range (box) and data range or 1.5× interquartile range (whisker). **e**, **g** Examples of two IC-lncRNAs closely related to immune cell infiltration. Fitting curve was performed by lm function. **h** The plot shows the odds ratios in each cancer type. The significance of overlap between lncRNAs differentially expressed between the high or low immune cell infiltration group and IC-lncRNAs co-regulating T-cell-related functions was calculated by two-sided Fisher’s exact tests. The n number was displayed in Supplementary Data 4. *p < 0.05, **p < 0.01, ***p < 0.001. The error bars were 95% confidence levels of odds ratios. The differentially expressed lncRNAs were calculated by fold-change and *t* test (FC > 2 or FC < 0.5, FDR < 0.05). **i** The plot shows the expression of significantly differentially expressed T-cell-related IC-lncRNAs between the high T-cell infiltration group and the low T-cell infiltration group.

Tumor-immune microenvironment is broadly populated with immune cells^29^. Therefore, we reasoned that if these IC-lncRNAs participate in tumor-immune microenvironment regulation, then they would be more likely to be highly expressed in immune cells and to be correlated with immune cell infiltration in tumors. Firstly, we found that a significantly higher proportion of IC-lncRNAs (90.7%) is expressed in immune cells by analyzing the immune cells RNA-seq datasets (Supplementary Note 1 and Fig. 4a, two-sided fisher’s exact test, *p* = 8.08e-92). In particular, 93.5% of the IC-lncRNAs co-regulating B cell-related functions are expressed in B cells. Moreover, we also found that the IC-lncRNAs co-regulating T cell-related functions are significantly more highly expressed than other lncRNAs in T cells, and the B cell-related IC-lncRNAs exhibit significantly higher expression in B cells (Fig. 4b, two-sided Wilcoxon-Mann-Whitney test, B cells *p* = 1.32e-10, T cells *p* = 2.67e-33). The results suggest that IC-lncRNAs exhibit higher expression in immune cell populations. All these results are also found in the single-cell RNA-seq dataset (Supplementary Note 2, Supplementary Fig. 11a and 11b).

We next estimated the associations between the expression of cooperative lncRNAs and infiltration levels of six immune cells (B cells, CD4 T cells, CD8 T cells, macrophages, neutrophils, and dendritic cells) in each cancer. We found that a large number of cooperative lncRNAs are correlated with immune cells infiltration in most cancers, such as breast invasive carcinoma (BRCA), LGG, LUSC, SKCM, and thyroid carcinoma (THCA) (Fig. 4c). In particular, 56.3% and 50.2% of cooperative lncRNAs are correlated with CD8 T-cell infiltration in LUSC and in SKCM respectively. The correlations between the expression of IC-lncRNAs co-regulating B-cell-related functions and B cell infiltration are significantly higher than others in LUSC (Fig. 4d, two-sided Wilcoxon-Mann-Whitney test, B cell *p* = 1.16e-12, CD4 T cell *p* = 7.00e-12, CD8 T cell *p* = 2.72e-20). For example, the *PCED1B-AS1* co-regulates B-cell activation with 8 IC-lncRNAs and the correlation coefficient between the expression and B-cell infiltration is as high as 0.69 (Fig. 4e). *PCED1B-AS1* could govern aerobic glycolysis, and its overexpression is closely related to larger tumor size and poorer survival^30^. In addition, the correlations between the expression of IC-lncRNAs co-regulating T cell-related functions and T-cell infiltration are significantly higher than others in SKCM, such as *RP11-861A13*, *RP11-25K19* and *LINC00324* (Fig. 4f, two-sided Wilcoxon-Mann-Whitney test, CD4 T cell *p* = 3.75e-05, CD8 T cell *p* = 2.22e-03). *LINC00324* co-regulating T-cell proliferation, activation, and differentiation are correlated with CD4 and CD8 T-cell infiltration with correlation coefficients 0.42 and 0.45, respectively (Fig. 4g). Indeed, *LINC00324* has been found to be overexpressed in cancer and correlated with aggressive cancer progress^31^.

We further used fisher’s exact test to investigate whether the IC-lncRNAs are likely to be associated with immune cell infiltration. The infiltration levels of CD8 T cells and CD4 T cells were used to divide the patients into two groups, respectively (high immune cell infiltration group and low immune cell infiltration group), and it was found that a lot of IC-lncRNAs co-regulating the T-cell functions are differentially expressed in majority of cancer types (Fig. 4h, two-sided fisher’s exact test). For example, *AP001055* and *CTD-2587H19* which regulate T cell-related functions are significantly highly expressed in LUSC patients with high CD4 T-cell infiltration (Two-sided *t* test, *AP001055* FDR = 1.32e-05, FC = 2.05; *CTD-2587H19* FDR = 1.04e-05, FC = 2.35) (Fig. 4i). In SKCM, *RP11-861A13* has significantly higher expression in the high CD8 T-cell infiltration group (Two-sided *t* test, FDR = 1.29e-07, FC = 2.17) (Fig. 4i). In summary, these results suggest that IC-lncRNAs exhibit higher expression in immune cells and are associated with immune cell infiltration, further validating the roles of the IC-lncRNAs in tumor-immune microenvironment.

### IC-lncRNA cooperation reveals similar regulation of cancer immune microenvironment

**Fig. 5:**
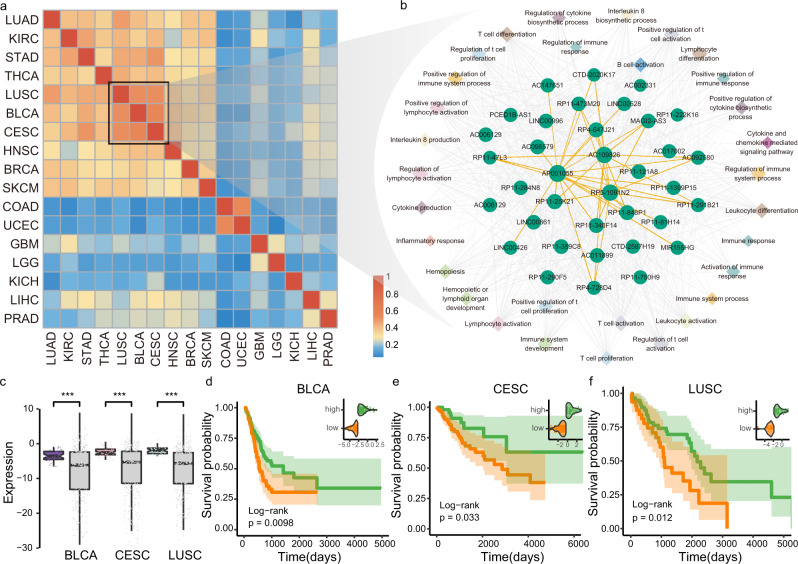
IC-lncRNA cooperation reveals similar regulation of cancer immune microenvironment. **a** Cancer clusters identified by hierarchical clustering based on Jaccard coefficients, calculated by the number of shared IC-lncRNAs divided by the size of the union set, plus expression Pearson correlations of pan-cancer IC-lncRNAs divided by 2. **b** The sub-network consisting of IC-lncRNAs shared by BLCA, CESC and LUSC and co-regulated functions. A circle shows an IC-lncRNA and a diamond shows an immune function. The same color diamond shows immune functions belonging to the same function category. The size of nodes indicates the number of connected nodes. An edge in orange represents a cooperative regulation between IC-lncRNAs; an edge in gray represents a regulation between IC-lncRNAs and immune functions. **c** The expression of IC-lncRNAs shared by BLCA, CESC, and LUSC (*n* = 36). The gray box shows the expression of other lncRNAs in each cancer. Two-sided *t* test was used. ***p < 0.001. The boxplots are shown as median (line), interquartile range (box) and data range or 1.5× interquartile range (whisker). **d**–**f** The difference in survival time between groups classified by the expression of the survival-related IC-lncRNAs identified by univariate cox regression in cancers was performed by log-rank test. The cutoff was defined by R function surv_cutpoint. The Kaplan-Meier plot of survival between two groups divided by the expression of *RP11-473M20* in BLCA (*n* = 246 samples), *MIR155HG* in CESC (*n* = 189 samples), and LUSC (*n* = 90 samples).

Several lines of evidence have indicated that sharing molecular features can reveal similar carcinogenic mechanisms between cancer types^32–34^. Here, we hypothesized that if the cancer types exhibit more similar IC-lncRNA cooperation patterns, they are more likely to be with similar immune mechanisms. Then, we computed a paired similarity score based on integrating the expression of IC-lncRNAs and the structure of LICNs in each cancer (Methods). We found that some cancers show greater similarity to each other than to other cancers, such as LUSC, BLCA, CESC, head, and neck squamous cell carcinoma (HNSC), BRCA, and SKCM (Fig. 5a and Supplementary Note 2, Supplementary Fig. 12-14), in which a general immune-related trend has emerged^35^. Especially, LUSC, BLCA, and CESC are closely clustered together. 41.2% (73/177) IC-lncRNAs are shared by at least two cancers (Supplementary Fig. 15). We focused on IC-lncRNAs that are common for the three cancers (Fig. 5b). We found that these IC-lncRNAs are widely involved in the co-regulation of immune-related functions, such as leukocyte activation, lymphocyte activation, and T-cell differentiation. We further discovered that the expression of shared IC-lncRNAs is significantly higher than other lncRNAs in three cancers (Fig. 5c, two-sided *t* test, all *p* < 9.43e-22). Both lung cancer and bladder cancer have shown relative effectiveness under immunotherapy using checkpoint blockade^36^. These observations suggest that IC-lncRNA cooperations might operate in cancer types with similar immune mechanisms.

Numerous studies elucidate how various components of the immune system control or contribute to cancer progression, thus revealing their prognostic value^37^. We next investigated whether these IC-lncRNAs are associated with the survival of cancer patients. Using the expression of shared IC-lncRNAs, cancer samples were optimally classified into two groups with significantly different overall survival rates respectively in all three cancers (Fig. 5d–f, log-rank test). For example, *MIR155HG* is significantly down-regulated in high-risk group as a protective factor, hazard ratio of which is 0.77 in LUSC. It has been shown that the expression of *MIR155HG* could predict overall survival in multiple cancers^38^. The approach based on immune lncRNA-lncRNA cooperation could effectively identify prognostic biomarkers. Collectively, these results suggest that the cooperative pattern based on both the structure of LICNs and the expression of IC-lncRNAs could reveal new cancer clusters from immune view.

### SKCM subtypes identified by IC-lncRNAs display different immune context and prognosis

**Fig. 6:**
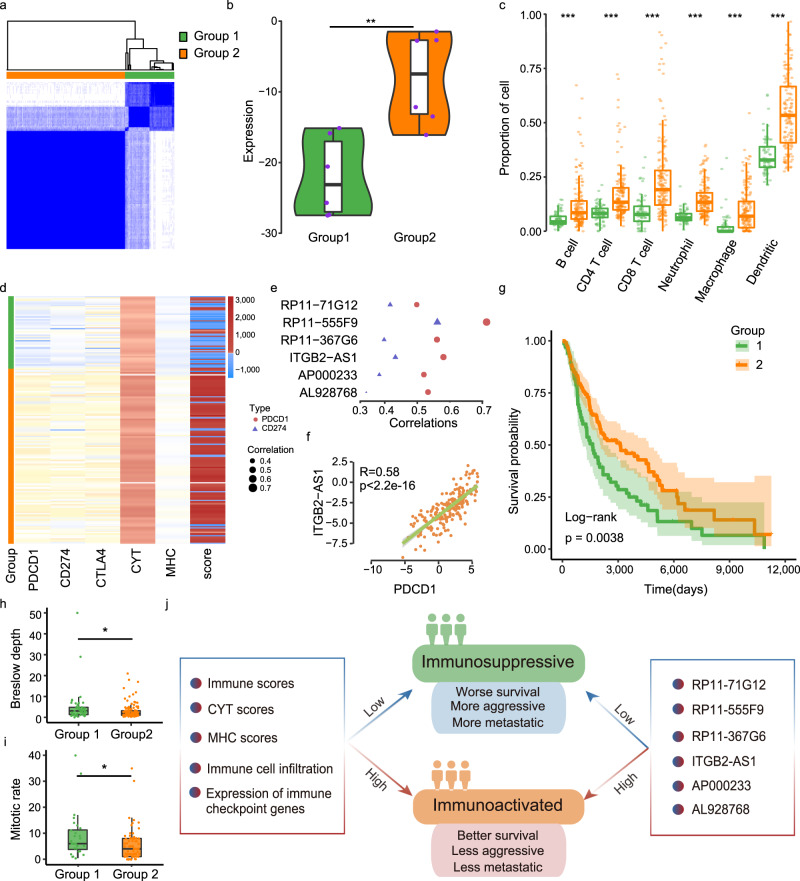
SKCM subtypes identified by IC-lncRNAs display different immune context and prognosis. **a** Two immune subtypes identified by expression of six immune hub IC-lncRNAs in SKCM. **b** The expression of the six IC-lncRNAs in two immune subtypes. The difference was calculated by two-sided *t* test (group 1 = 66 samples, group 2 = 159 samples). **p < 0.01. The boxplots and the violin plots are shown as median (line), interquartile range (box), and data range or 1.5× interquartile range (whisker), each point indicates an IC-lncRNA. **c** The infiltration levels of six immune cells in two immune subtypes. Wilcoxon-Mann-Whitney test was used (*n* = 225 samples). ***p < 0.001. The boxplots are shown as median (line), interquartile range (box), and data range or 1.5× interquartile range (whisker), each point indicates immune cell infiltration of a sample. **d** The distribution of immune indicators (CYT, MHC, and score (immune score)) and expression of immune checkpoint genes *PDCD1, CD274, CTLA4* in two immune subtypes. **e** Pearson’s correlations between the expression of these IC-lncRNAs and *PDCD1* or *CD274*. All p < 0.05. **f** The expression of IC-lncRNA *ITGB2****-****AS1* is closely related to *PDCD1*. Fitting curve was performed by lm function. **g** Kaplan-Meier plot of survival for two immune subtypes in SKCM (*n* = 224 samples). The survival difference is calculated by log-rank test. **h** The distribution of Breslow’s depth in two subtypes of SKCM (*n* = 166 samples). The boxplots are shown as median (line), interquartile range (box), and data range or 1.5× interquartile range (whisker), each point indicates Breslow’s depth of a sample. **i** The distribution of mitotic rate in two subtypes of SKCM (*n* = 98 samples). The boxplots are shown as median (line), interquartile range (box), and data range or 1.5× interquartile range (whisker), each point indicates mitotic rate of a sample. Difference in **h** and **i** was calculated by two-sided Wilcoxon–Mann–Whitney test. *p < 0.05. **j** An illustration of two immune subtypes in SKCM. The immunoactivated subtype is characterized by higher immune cell infiltration levels, higher expression of immune indicators and immune checkpoint genes, better survival, and wider invasion and metastasis. In contrast, the immunosuppressed subtype has the opposite phenotype.

The skin cutaneous melanoma is a malignancy of melanocytes, which accounts for the most number of deaths from skin cancer^39^. Therefore, we investigated to what extent the IC-lncRNAs can be applied to SKCM molecular subtyping. We first identified the top 10% IC-lncRNAs with larger expression fluctuation and further filtered by hubs in SKCM. Finally, 6 IC-lncRNAs (*RP11-71G12*, *RP11-555F9*, *RP11-367G6*, *ITGB2-AS1*, *AP000233* and *AL928768*) were obtained. These 6 IC-lncRNAs participate in immune functions such as regulation of B-cell activation, immune response, lymphocyte activation, and immune system process. These IC-lncRNAs are correlated with immune cell infiltration and they are also more highly expressed in B cells (Supplementary Fig. 16a, b, two-sided Wilcoxon-Mann-Whitney test, B cell *p* = 0.0015). Next, we found that the SKCM patients can be classified into two subtypes based on the expression of the 6 IC-lncRNAs (Fig. 6a). These IC-lncRNAs are highly expressed in group 2 patients (Fig. 6b, two-sided *t* test, *p* = 2.81e-3, Supplementary Fig. 17). There are no significant differences in age, gender, and cancer stages observed between the two subtypes (Supplementary Fig. 17). We further compared six immune cell infiltration levels between two subtypes respectively. We found that group 2 patients consistently have significantly higher infiltration levels than group 1 patients for all immune cells (Fig. 6c, two-sided Wilcoxon-Mann-Whitney test, all *p* < 5.65e-09). Particularly, the average infiltration level of CD8 T cells in group 2 is 25%, while the infiltration level is only 9% in group 1. At the same time, the average infiltration level of dendritic cells (DC) of group 2 patients is almost double that in group 1. It has been shown that the presence of mature DC within tumors is a positive prognostic factor in melanoma patients and melanoma-associated DCs is also related to immunotherapy^40^.

It has been demonstrated that some indicators are useful biomarkers for predicting the immune response, such as immune cytolytic activity (CYT) and major histocompatibility complex (MHC) scores and the immune score^41,42^. We further investigated the distribution of these scores among SKCM patients. We found that group 2 patients have significantly higher CYT, MHC, and immune scores than group 1 (Fig. 6d, two-sided Wilcoxon-Mann-Whitney test, all *p* < 9.46e-12) Treatment with immune checkpoint blockade has transformed the outcome for patients with melanoma, such as programmed cell death protein 1 (PDCD1) inhibitors, CTLA4 inhibitor^43^. Therefore, we compared the expression of immune checkpoint genes between two subgroups (Supplementary Fig. 18). We found that *PDCD1*, *CD274,* and *CTLA4* are significantly higher expressed in group 2 (Fig. 6d, two-sided Wilcoxon-Mann-Whitney test, all *p* < 4.29e-07). At the same time, the expression of all the six IC-lncRNAs is significantly positively correlated with most immune checkpoint genes, such as *PDCD1* and *CD274* (Supplementary Fig. 19 and Fig. 6e, Pearson correlation, *p* < 0.05). Each IC-lncRNA is more closely related to *PDCD2* than *CD274* in expression. In particular, the correlation coefficient between IC-lncRNA *ITGB2-AS1* and *PDCD1* is as high as 0.58 (Fig. 6f). IC-lncRNA *ITGB2-AS1* is antisense to *ITGB2* and has been shown to be highly expressed, and closely related to immunosuppression in AML^44^. *ITGB2-AS1* may be used as a potential immunotherapeutic target in cancers.

The degree of tumor infiltration by immune cells can predict a patient’s clinical outcome in many cancer types^45,46^. Therefore, we explored the prognostic implications of molecular subgroups. We found that patients in group 2 have significantly better overall survival than group 1 (Fig. 6g, log-rank test *p* = 3.8e-03). We also further investigated the invasion and metastasis of these SKCM patients. We found that group 1 patients have significantly higher Breslow’s depth and mitotic count than group 2 (Fig. 6h, *p* = 0.012 and Fig. 6i, *p* = 0.022, two-sided Wilcoxon-Mann-Whitney test). Breslow’s depth is a description of how deeply tumor cells have invaded and the higher the mitotic count, the more likely the tumor is to have metastasized^47^. It has been shown that the two indicators are important prognostic factors, the higher the two indicators, the worse the prognosis is^48,49^. These results suggest that IC-lncRNAs identify different SKCM subtypes (group 1 immunosuppressive subtype and group 2 immunoactivated subtype) with remarkable immunology diversity (Fig. 6j), which is helpful to improve personalized cancer management.

## Discussion

Accumulating evidence suggests that lncRNAs could crosstalk each other by co-regulating biological functions. However, systematic analysis of lncRNA co-regulation is lacking especially in cancer. In the current study, we provided a multiple-step model to identify lncRNA-lncRNA cooperation based on co-regulating functional modules by integrating multi-omics data. In this model, cancer-context lncRNA-target pairs were first identified by a multivariate linear model, which factored in variation (noise) in mRNA expression induced by the expression of lncRNA, changes in DNA copy number, and promoter methylation. We did observe that some target genes of lncRNAs are significantly affected by copy number and methylation, and these effects are distributed differently among cancers (Supplementary Fig. 20). Furthermore, 99% of these target genes are positively regulated by copy number. Among the target genes significantly regulated by methylation, most of them are negatively modulated by methylation. Then, lncRNA cooperation was detected via functional modules based on lncRNA-target pairs data, which allowed for an in-depth analysis of individual lncRNAs in the context of their cooperative surroundings in cancer. Systematic construction and analysis of lncRNA-lncRNA cooperation networks across multiple cancers can help to elucidate the commonalities and differences in cancer mechanisms.

Recently, mounting studies have found that lncRNAs play roles in tumor-immune microenvironment^4^. However, how lncRNAs contribute to tumor immunity is still known little. We found that lncRNAs tend to co-regulate a wide range of immune-related functions, especially T-cell activation. T cells play critical roles in host defense against cancer^50^. The aim of cancer immunotherapy is to promote the activity of T cells within a tumor and establish efficient and durable antitumor immunity^51^. IC-LncRNAs co-regulating T-cell activation may provide new views for cancer immunotherapy. At the same times, we found some cancer clusters with similar immune lncRNA co-regulation mechanism. Countless patients with a broad range of tumor types have seen pronounced clinical effects with immunotherapeutic intervention; however, patients of different cancer types have different responses when provided the same treatment^29^. Diversity of the immune regulation in tumor may influence effects of immunotherapy, and cancer clusters based on immune lncRNA co-regulation could provide evidence for clinical tumor immunotherapy in future.

The crosstalk between cancer and the immune system is very complex. T cells can target tumor cells in various ways, either directly by eliminating tumor cells or indirectly by modulating the tumor-immune microenvironment^51^. Particularly, interactions between ligands and activating or inhibitory receptors are crucial for further regulating T-cell activation and tolerance^52^. We found that some IC-lncRNAs could target proteins mediating interactions between T cells and tumor cells such as *LINC00324*, *RP11-445H22,* and *RP11-25K19*. *RP11-445H22* could positively regulate *CTLA4* and *PDCD1* both of which are inhibitory receptors of T cells in LUAD. In addition, *RP11-25K19* could also positively regulate *CXCR3* and *CXCL10* which are activating receptors of T cells in SKCM. These IC-lncRNAs may be as cancer-immune therapeutic targets.

We systematically analyzed lncRNA immune co-regulations in pan-cancer, and found that the potential clinical application of IC-lncRNAs is different among cancer types. Melanoma is a highly aggressive form of skin cancer, where it is often difficult to treat with traditional therapies^40^. The long-term prognosis of metastatic melanoma is poor and prognosis biomarkers remain elusive. We found that IC-lncRNAs have the most significant contribution to tumor typing in SKCM. The two immune-related subtypes of SKCM display obvious diversity in the immune context of the tumor microenvironment, invasion, metastasis, and survival time. The IC-lncRNAs cannot be used to identify lung cancer subtypes, while several IC-lncRNAs have prognostic potential in lung cancer. In LUSC, 3 IC-lncRNAs are protective factors that are highly expressed in the low-risk group, and other one IC-lncRNA acted as a risky factor of which increased expression is associated with a poor survival outcome (Supplementary Fig. 21). It is suggested that IC-lncRNAs play a role in tumor-immune regulation with a cancer-context-specific way. It is valuable to further determine the molecular mechanism of each lncRNA immune co-regulation.

In summary, we presented the lncRNA cooperation landscape across human major cancers and showed the importance of immunity. Our study opens new avenues to investigate the functions and mechanisms of lncRNAs in tumor-immune microenvironment. Follow-up investigation is warranted to deepen our understanding of lncRNAs cancer immune functions and their application in clinics.

## Methods

### Omics data across cancer types

The paired lncRNA-mRNA expression profiles as well as DNA methylation and copy number data were obtained from the TCGA (https://portal.gdc.cancer.gov/)^53^. The mRNA expression datasets profiled via RNA sequencing (level 3, TPM) were used. LncRNA expression datasets (FPKM) were obtained from The Atlas of Non-coding RNAs in Cancer (TANRIC, https://www.tanric.org/)^54^. The expression values of mRNAs and lncRNAs were log2 transformed for subsequent analysis. The methylation level for genes was defined as the average beta-values of probes mapping to the corresponding gene promoter. In addition, DNA copy number datasets were downloaded from Firehose (https://gdac.broadinstitute.org/)^55^. In total, each cancer type had paired lncRNA and mRNA expression profiles as well as DNA methylation and copy number data measured for the same sample, forming a pan-cancer data compendium from 5284 tumor samples for 17039 mRNAs and 12727 lncRNAs (Supplementary Table 1). The clinical information of patients, including the survival status, stage, age, and survival time, was also downloaded from the TCGA project (Supplementary Note 1).

### Identification of lncRNA-lncRNA cooperation across cancer types

We proposed a multiple-step model to identify lncRNA-lncRNA functional cooperation in each cancer type. First, a multivariate linear regression model was used to identify cancer-context lncRNA-target regulations. Then, the cooperative lncRNA pairs were identified as follows: lncRNA pairs that significantly shared target genes were initially identified; for each significantly lncRNA pair, we identified candidate functional modules based on the shared targets; and then, the candidate modules were further filtered using two topological features in the protein-protein interaction network. Here, a pair of lncRNAs were defined as cooperative if they significantly co-regulated at least one functional module.

#### Prediction of cancer-context lncRNA-target regulation

The multivariable linear regression model was used to assess the association between the expression of lncRNA and mRNA in each cancer type and considered the effect of methylation and DNA copy number on the expression of mRNAs. For each pair of lncRNA and mRNA, the predicted model was defined as follows:

1 $${{\it{y}}}_{{{{{{\rm{j}}}}}}}={{{\beta }}}_{0}+{{{\beta }}}_{{{{{{\rm{CN}}}}}},{{{{{\rm{j}}}}}}}\times {{\it{x}}}_{{{{{{\rm{CN}}}}}},{{{{{\rm{j}}}}}}}+{{{\beta }}}_{{{{{{\rm{Me}}}}}},{{{{{\rm{j}}}}}}}\times {{\it{x}}}_{{{{{{\rm{Me}}}}}},{{{{{\rm{j}}}}}}}+{{{\beta }}}_{{{{{{\rm{\mu }}}}}},{{{{{\rm{j}}}}}}}\times {{\it{x}}}_{{{{{{\rm{\mu }}}}}},{{{{{\rm{j}}}}}}}+{{\varepsilon }}$$

Where *y*_j_ is the expression of mRNA_j_, *x*_CN_, *x*_Me_, *x*_*μ*_ are CNV and promoter methylation at mRNA_j_ and the expression of lncRNA_μ_ respectively, *β*_0_ is an intercept and *ε* is a random error. The lncRNA-target pairs with FDR <0.01 were further filtered with Bonferroni-corrected *p* value <0.1.

#### Identification of functionally cooperative lncRNA pairs

We used the method proposed in our previous study to identify the functionally lncRNA-lncRNA cooperations^56^, of which briefly steps are as follows: first, the lncRNA pairs sharing at least three target genes were identified as candidates. Then, shared target genes of each candidate lncRNAs pair were used to identify functional modules. The shared targets were performed functional enrichment by hypergeometric test across selected GO terms. Thus, at a given significant level, we can achieve enriched GO terms and a subset of shared targets annotated to each of these GO terms as a candidate functional module. Next, two topological features were further used to filter the candidate module in the protein interaction network: (i) the minimum distance from each gene to others in the subset is no larger than 2. (ii) the characteristic path length (CPL) is significantly shorter than random. 1000 random networks were created using the edge-switch method with the software Mfinder (available at http://www.weizmann.ac.il/mcb/UriAlon/). We defined the *p* value as the fraction of CPL for the same subset that was shorter than that in the real network. Finally, after performing function enrichment and two topological restrictions in the network, a pair of lncRNAs were considered cooperative if they co-regulated at least one functional module.

### Correlations between lncRNAs and immune cell infiltration

The infiltration proportion of B cells, CD4 T cells, CD8 T cells, macrophages, neutrophils, and dendritic cells were predicted by TIMER (http://cistrome.shinyapps.io/timer), respectively^57^. Spearman’s rank correlation was calculated between the expression of each lncRNAs and infiltration proportion of immune cells. Wilcoxon-Mann-Whitney test was used to evaluate the difference of correlation between IC-lncRNAs co-regulating corresponding immune cell-related functions and other lncRNAs with immune cell infiltration. In addition, we classified the patients into two groups according to the median infiltration proportion of CD4 T cell and CD8 T cell respectively in each cancer. Differentially expressed lncRNAs between the two groups were identified using fold-change and *t* test (FC > 2 or FC < 0.5 and FDR < 0.05). Fisher’s exact test was used to evaluate the significant overlap between differentially expressed lncRNAs and IC-lncRNAs co-regulating T cell-related functions.

### Evaluation of similarity among cancers based on LICNs

We evaluated the degree of similarity between cancers based on both the structure of LICNs and the expression of immune cooperative lncRNAs. First, we calculated Jaccard coefficient of IC-lncRNAs in LICNs between any two cancers as R1. Then, Pearson’s correlation coefficient was used to compare the expression of IC-lncRNAs in any two cancers as R2. At last, Hierarchical clustering was used to identify cancer clusters with similar immune mechanisms based on the mean values of R1 and R2.

### SKCM subtype classification based on IC-lncRNAs

IC-lncRNAs in SKCM were firstly filtered with expression value >0 in larger than 50% of samples. Then, we selected the top 10% of IC-lncRNAs according to the variance of expression in descending order and further filtered by hubs. We lastly identified two SKCM subtypes based on this IC-lncRNAs expression by using ConsensusClusterPlus R package^58^ (Supplementary Note 1).

### Survival analysis of IC-lncRNAs in LUSC

A univariate cox regression analysis was used to evaluate the association between survival time and the expression of each IC-lncRNA in LUSC. The candidate prognostic IC-lncRNAs were identified by *p* values <0.05. A multivariate cox regression analysis and bidirectional elimination were performed to find prognostic IC-lncRNA signatures. We assigned a risk score to each patient taking into account both the strength and positive or negative association of each lncRNA with survival. A risk score per sample, according to a linear combination of the IC-lncRNA expression weighted by the regression coefficients from multivariate cox regression analysis, was defined as follows:

2 $${{{{{\rm{Risk}}}}}}\;{{{{{\rm{score}}}}}}=\mathop{\sum}\limits_{{\it{j}}=1}^{{\it{n}}}{{{\beta }}}_{{{{{{\rm{j}}}}}}}\times {{{{{{\rm{exp}}}}}}}_{{\it{j}}}$$

Where n is the number of IC-lncRNAs, *β*_j_ is the regression coefficients of IC-lncRNA_*j*_ and exp_*j*_ is the expression of lncRNA_j_. Then, patients were divided into a high-risk group and a low-risk group according to the median value of the risk score. The Kaplan-Meier method was used to estimate the overall survival for the two groups, and differences in survival were analyzed using the log–rank test.

### Statistics and reproducibility

The statistical significance of differences between groups was evaluated using Wilcoxon-Mann-Whitney test and *t* test. Fisher’s exact test was used to determine whether or not there was a significant association between two categorical variables. The survival difference between groups was assessed by log-rank test. The sample sizes for each cancer were displayed in Supplementary Table 1. A *p* value < 0.05 was considered significant (*p < 0.05; **p < 0.01; ***p < 0.001; ****p < 0.0001). The statistical analyses were performed using RStudio (Version 1.3.1093) with R software version 3.6.3 and 4.0.3.

### Reporting summary

Further information on research design is available in the Nature Portfolio Reporting Summary linked to this article.

## Supplementary information

Supplementary Information

Description of Additional Supplementary Data

Dataset 1

Dataset 2

Dataset 3

Dataset 4

Reporting Summary

## Data Availability

The paired lncRNA-mRNA expression profiles as well as DNA methylation and copy number data were obtained from the TCGA Data Portal. The RNA-seq datasets of immune cells were downloaded from GEO (https://www.ncbi.nlm.nih.gov/geo/) under accession number GSE26530, GSE30811, GSE33772, GSE34260, GSE36952, GSE40131, GSE40548, GSE40718, GSE45734, GSE45982, GSE53419, GSE55320, GSE55536, GSE56179, GSE57494, GSE58596, GSE59846, GSE60482, GSE64182, GSE64655, GSE64713, GSE66117, GSE66385, GSE66763, GSE66895, GSE68482, GSE68795, and GSE72502. The Biological Process (BP) terms for Gene Ontology (GO) were downloaded from the MSigDB (v5.1) database (https://www.gsea-msigdb.org/gsea/msigdb/index.jsp). Cancer-related lncRNAs were obtained from lnc2cancer 3.0 (http://bio-bigdata.hrbmu.edu.cn/lnc2cancer/) and literatures (Supplementary Data 2). The source data used to generate the main figures is provided in Supplementary Data 4.

## Code availability

The code used in the work is available on GitHub (https://github.com/yunjinxie/stt-lncRNA2022) with the identifier (10.5281/zenodo.7275958). All software tools used in this study are freely available.
