# Supplementary material for: Surveying lncRNA-lncRNA cooperations reveals dominant effect on tumor immunity cross cancers
Source: Commun Biol. 2022 Dec 3;5:1324. doi: 10.1038/s42003-022-04249-0 (PMC9719535; doi:10.1038/s42003-022-04249-0)
Supplement: Supplementary file 1 — Supplementary Information [file 42003_2022_4249_MOESM1_ESM.pdf]

Surveying lncRNA-lncRNA cooperations reveals dominant effect on tumor immunity cross cancers

Tingting Shao[1, #], Yunjin Xie[1, #], Jingyi Shi[1, #], Changbo Yang[1], Haozhe Zou[1], Yongsheng Li[2, *], Juan Xu[1, *], Xia Li[1,2 *]

[#]These authors contributed equally.

*To whom correspondence should be addressed.

Corresponding authors：

Xia Li, Email: lixia@hrbmu.edu.cn.

Juan Xu, Email: xujuanbiocc@ems.hrbmu.edu.cn.

Yongsheng Li, Email: liyongsheng@ems.hrbmu.edu.cn.

Supplementary information

(Supplementary Note 1, Supplementary Note 2, Supplementary Figure 1 – 21 and Supplementary Table 1).

Supplementary Note 1

Omics data across cancer types

All mRNA expression datasets were obtained from TCGA Data Portal[1] and profiled via RNA sequencing. For mRNA expression, mapped and gene-level-summarized (level 3, TPM) RNA-seq datasets were used. We further removed mRNAs with a read count less than 20 that were detected in > 95.0% of samples in each cancer type dataset. The expression values were log2 transformed for subsequent analysis.

lncRNA expression datasets were obtained from TANRIC[2]. We further removed lncRNA with FPKM less than 0.1 that were detected in > 95.0% of samples in each cancer type dataset. The expression values were log2 transformed for subsequent analysis.

DNA methylation datasets were also obtained from TCGA Data Portal (level 3), and the methylation values for each protein-coding gene were defined as the average beta-values of probes mapping to the corresponding gene promoter (±2 kb of annotated transcription start sites).

In addition, DNA copy number datasets were obtained from Firehose[3]. We used level 4 non-discretized gene-summarized log2-transformed aCGH copy-number calls (tumour/normal ratio) computed by the Gistic2 algorithm (ftp://ftp.broadinstitute.org/pub/GISTIC2.0/)[4].

Furthermore, Immune cells RNA-seq datasets were downloaded from GEO[5,6] (GSE26530[7,8], GSE30811[9], GSE33772[10], GSE34260[11], GSE36952[12,13], GSE40131[14], GSE40548[15,16], GSE40718[17], GSE45734[18], GSE45982[19,20], GSE53419, GSE55320[21], GSE55536[22], GSE56179[23], GSE57494[24], GSE58596[25], GSE59846[26], GSE60482[27], GSE64182[28], GSE64655[29], GSE64713[30], GSE66117[31], GSE66385[32], GSE66763[33], GSE66895[34], GSE68482[35], GSE68795[36], GSE72502[37]).

Single-cell RNA-seq data was downloaded from GEO (GSE75688[38]).

The immune checkpoint genes were obtained from the previous study[39].

Functional annotation of target genes

The Biological Process (BP) terms for Gene Ontology (GO) were downloaded from the MSigDB (v5.1) database[40]. As in previous studies, process categories from GO were restricted to BP terms such that the number of genes annotated to a term was at least 5 and no more than 500. Ultimately, 792 filtered GO BP terms were used for further analysis. Moreover, a list of GO terms determined to be related to the hallmarks of cancer were obtained from a previous study[41].

Protein-protein interaction network of target genes

We assembled protein-protein interaction data from HPRD (http://www.hprd.org/index_html)[42] and further removed self-loop interactions. Then, the gene symbols for each interaction were mapped to their corresponding Entrez gene identifiers. Last, the maximum component of the whole protein-protein interaction network was extracted, which contained 35,865 interactions among 9,028 genes.

Genomic location of cooperative lncRNAs

The starting point and the end point of co-regulated lncRNAs on chromosomes were obtained by mapping each lncRNA ensembl ID to reference genome from Gencode v19 (https://www.gencodegenes.org/). Then, we classified these cooperative lncRNAs pairs into three groups: (i) two lncRNAs located at different chromosomes. (ii) two lncRNAs located at the same chromosome with distance less than 10Mb. (iii) two lncRNAs located at the same chromosome with distance more than 10Mb. The p value was the fraction of the number of cooperative lncRNA pairs for the same group that was larger than that for random set by 1000 randomly sampling with equal number of lncRNA pairs from lncRNA expression profiles across cancer types.

Expression pattern of IC-lncRNAs and IC-lncRNA co-regulated pairs

To illustrate the expression pattern of immune cooperative IC-lncRNAs, we compared the mean expression of IC-lncRNAs with an equal number of non-IC lncRNAs randomly sampling from the expression profile in each cancer by t.test function. For each cancer, we created a dataset that included all IC-lncRNA pairs (class 0) and an equal number of non-IC-lncRNA pairs randomly sampling from the expression profile (class 1). Then, we calculated Pearson correlation of expression between a pair of lncRNAs in each class. We compared the correlations between two classes by t.test function.

Subtype classification of patients based on IC-lncRNAs.

We used the ConsensusClusterPlus R package to identify the optimum number of clusters in SKCM expression data[43]. We selected 80% sample resampling (pItem), a maximum evaluated k of 5 so that cluster counts of 2,3,4,5 are evaluated (maxK), 50 resamplings (reps), Hierarchical clustering (clusterAlg) upon euclidean distances (distance). We also specified a random seed in order to be repeatable. Since we found the number of samples in each cluster is not abundant when k is larger than 2, we finally classified samples into two subtypes according to relative change in area under CDF curve and tracking plot.

The cancer-associated lncRNAs and targets of anticancer drugs

Cancer-related lncRNAs were obtained from lnc2cancer 3.0[44] and literatures. All the targets of the US Food and Drug Administration (FDA) approved drugs were downloaded from the

DrugBank (https://go.drugbank.com/) database[45]. and anticancer drugs were obtained according to the Anatomical Therapeutic Chemical (ATC) classification system. If one drug had the first two levels of ATC code L01 (antineoplastic agents), this drug was considered an anticancer drug.

Supplementary Note 2

IC-lncRNAs were correlated with immune cell (B cells and T cells) infiltration.

We also investigated the expression of the IC-lncRNAs in immune cells by scRNA-seq dataset (GSE75688). We found that a significantly higher proportion of IC-lncRNAs (83.0%) is expressed in immune cells (Supplementary Note 1 and Supplementary Figure 11a, two-sided fisher's exact test, p=1.60e-91). In particular, 72.7% of the IC-lncRNAs co-regulating B cell-related functions are expressed in B cells. Moreover, we also found that the IC-lncRNAs co-regulating T cell-related functions are significantly more highly expressed than other lncRNAs in T cells and the B cell-related IC-lncRNAs exhibit significantly higher expression in B cells (Supplementary Figure 11b, two-sided Wilcoxon-Mann-Whitney test, B cells p=1.33e-03, T cells p=5.19e-08). The results suggest that IC-lncRNAs exhibit higher expression in immune cell populations.

IC-lncRNA cooperation reveals similar regulation of cancer immune microenvironment.

We also found that colon adenocarcinoma (COAD) and uterine corpus endometrial carcinoma (UCEC) are closely clustered together and their similarity in IC-lncRNA cooperative pattern is higher than other cancers (Figure 5a). The two cancers have four common IC-lncRNAs which are widely involved in co-regulation of immune-related functions, such as regulation of lymphocyte activation and differentiation (Supplementary Figure 12 and Supplementary Figure 13). We further found that the expression of shared cooperative IC-lncRNAs is significantly higher than other lncRNAs in two cancers (Supplementary Figure 14, two-sided t test, COAD p=0.0013, UCEC p=0.016).

Supplementary Figures

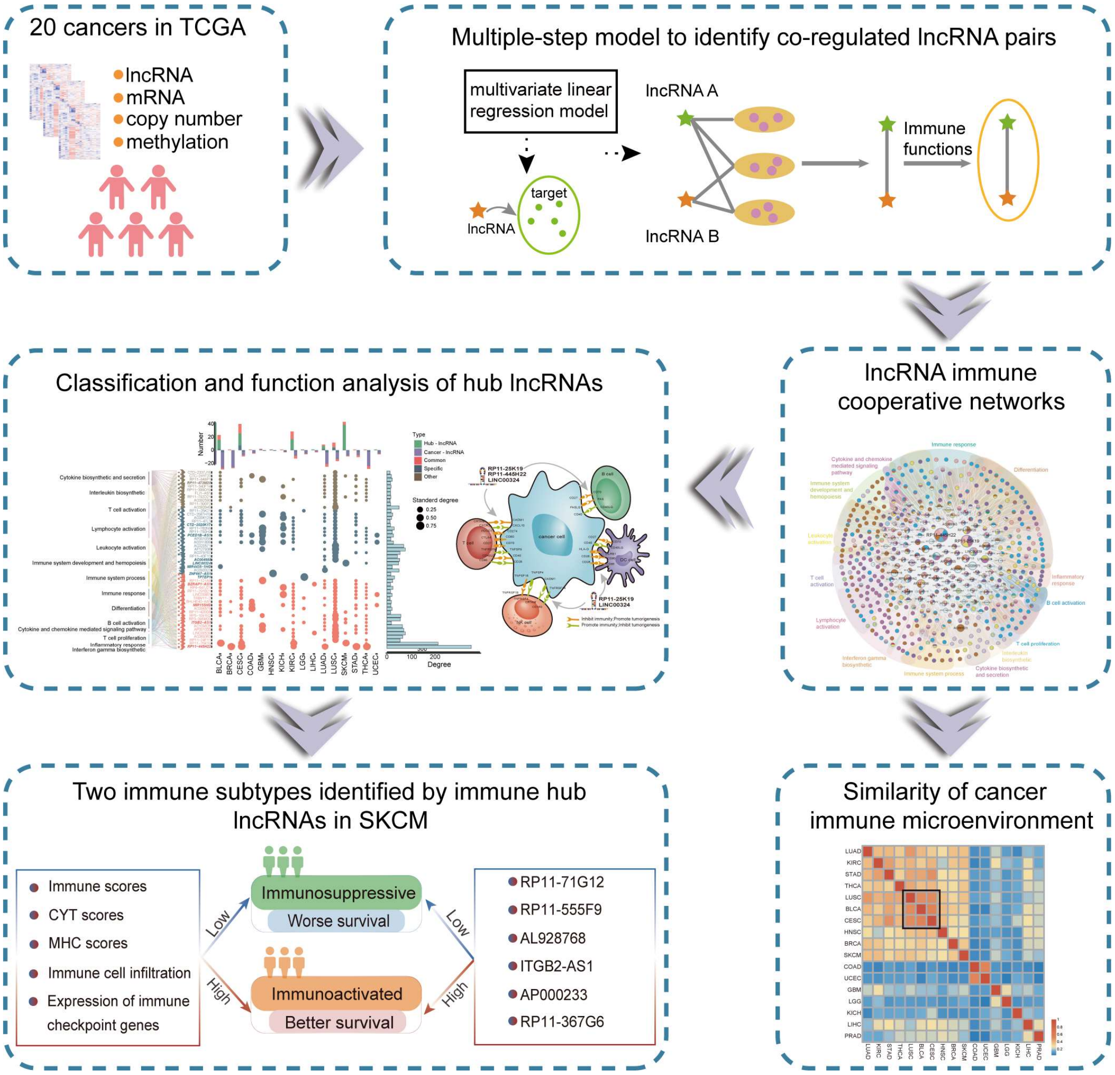

Supplementary Figure 1. The flowchart of the research. We first obtained lncRNA and mRNA expression profiles, copy number datasets, methylation datasets and clinical datasets of 20 cancers in TCGA. Then, we identified lncRNA-lncRNA cooperations by a multiple-step model. Next, a series of functional analysis were performed on cooperative lncRNAs and a lncRNA immune cooperative networks (LICNs) was constructed. Some hub IC-lncRNAs had various distributions in cancers and played important roles in cancer progression and tumor cell-immune cell communication. The similarity of cancer immune microenvironment can be revealed by IC-lncRNA cooperations. Finally, six IC-lncRNAs were identified as prognostic markers in SKCM by consensus clustering.

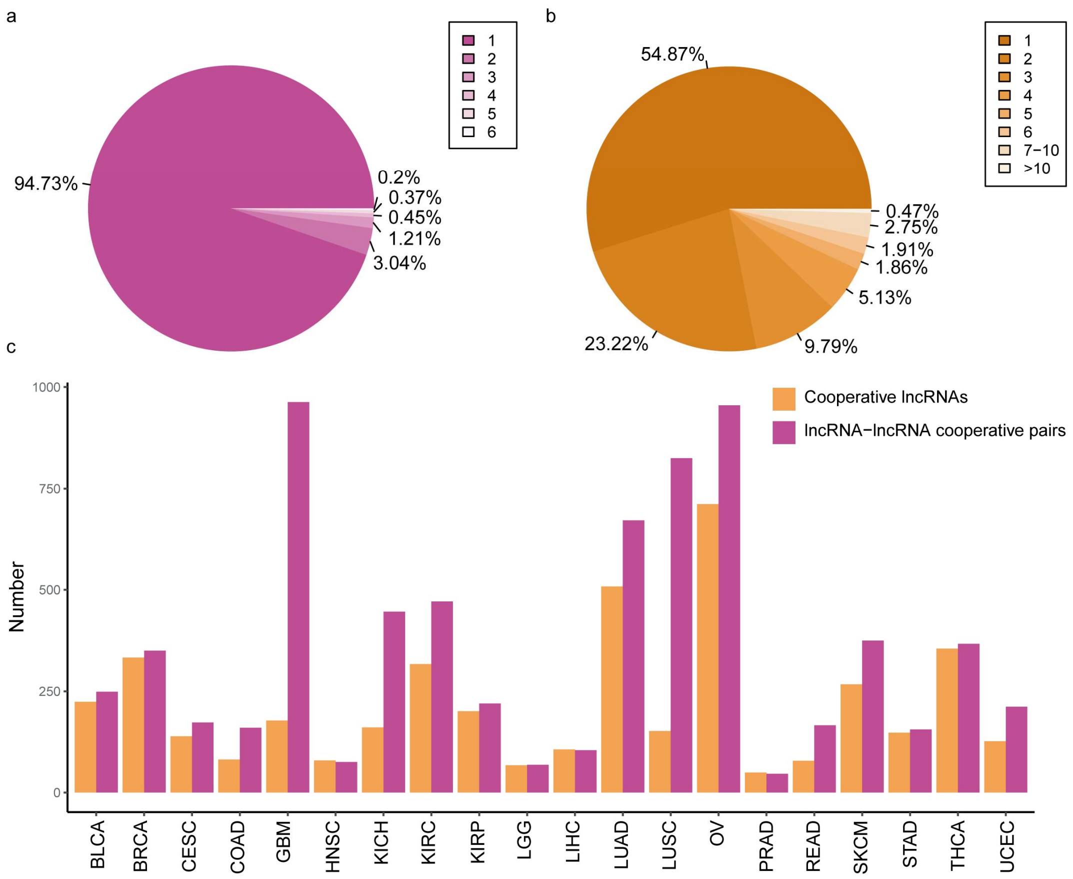

Supplementary Figure 2. The distribution of cooperative lncRNAs and lncRNA cooperative regulations across cancer types. (a) The pie chart shows the proportion of lncRNA-lncRNA cooperative regulations in different numbers of cancer types. **(b)** The pie chart shows the proportion of cooperative lncRNAs occurred in different numbers of cancer types. **(c)** The number of cooperative lncRNAs and lncRNA-lncRNA cooperative regulations in cancers.

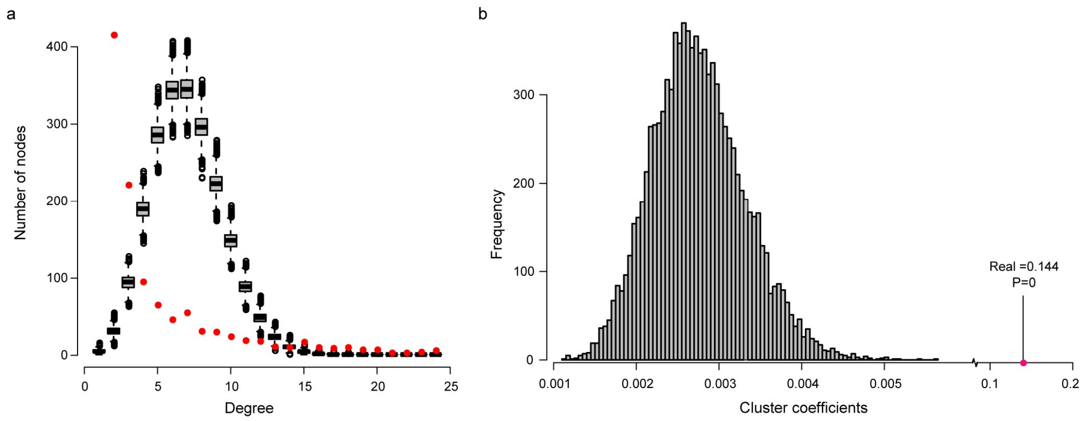

Supplementary Figure 3. The topological characters of pan-cancer lncRNA-lncRNA cooperative network. (a) Distribution of degrees for the observed pan-cancer lncRNA-lncRNA cooperative network (red circles) and permuted networks (box plots). **(b)** The cluster coefficient of the pan-cancer lncRNA-lncRNA cooperative network is higher than those of the random networks.

a b

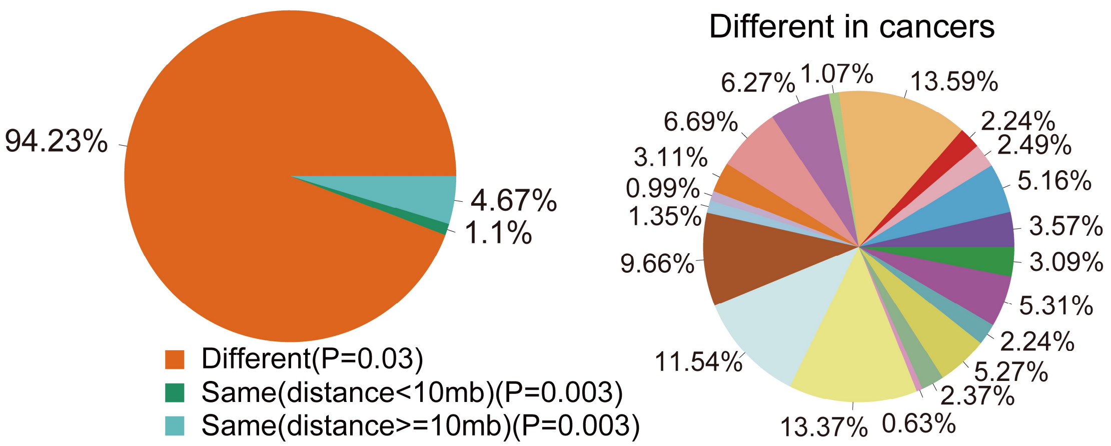

Supplementary Figure 4. Analysis of genomic location about cooperative lncRNAs. (a) The pie chart shows the proportion of cooperative lncRNA-lncRNA pairs located on different chromosomes, the same chromosome but the distance within 10 mb and the same chromosome but the distance more than 10 mb. **(b)** The pie chart shows the proportion of lncRNA pairs located on different chromosomes across cancer types. The p values are obtained by random test.

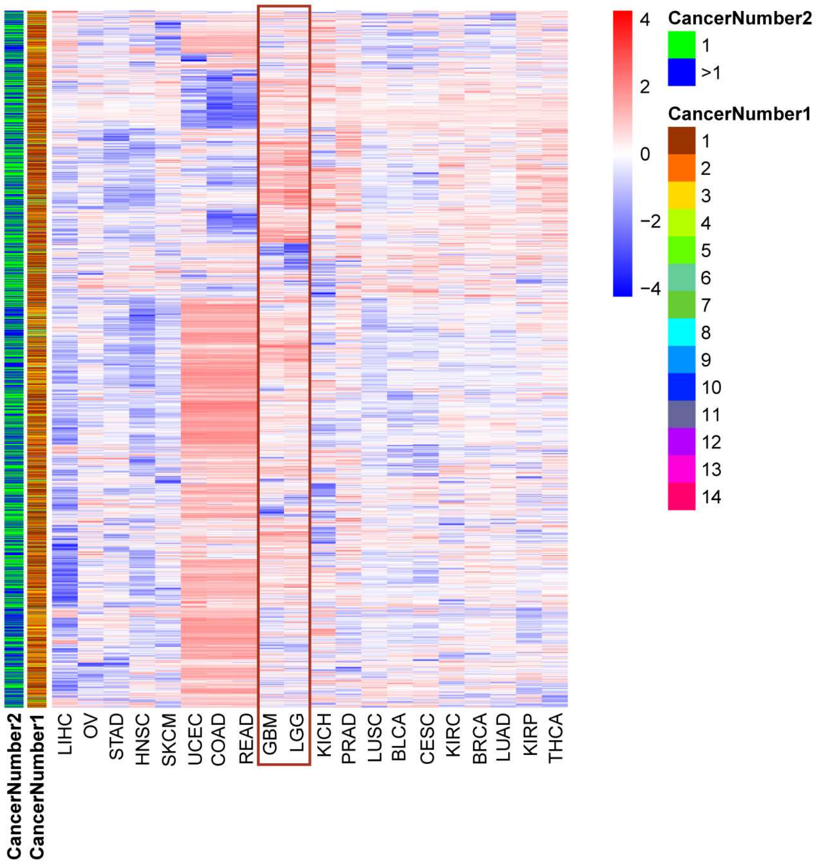

Supplementary Figure 5. The expression of cooperative lncRNAs across cancer types. The lncRNAs were classified into 14 categories accorrding to the number of cancer types in which they occurred.

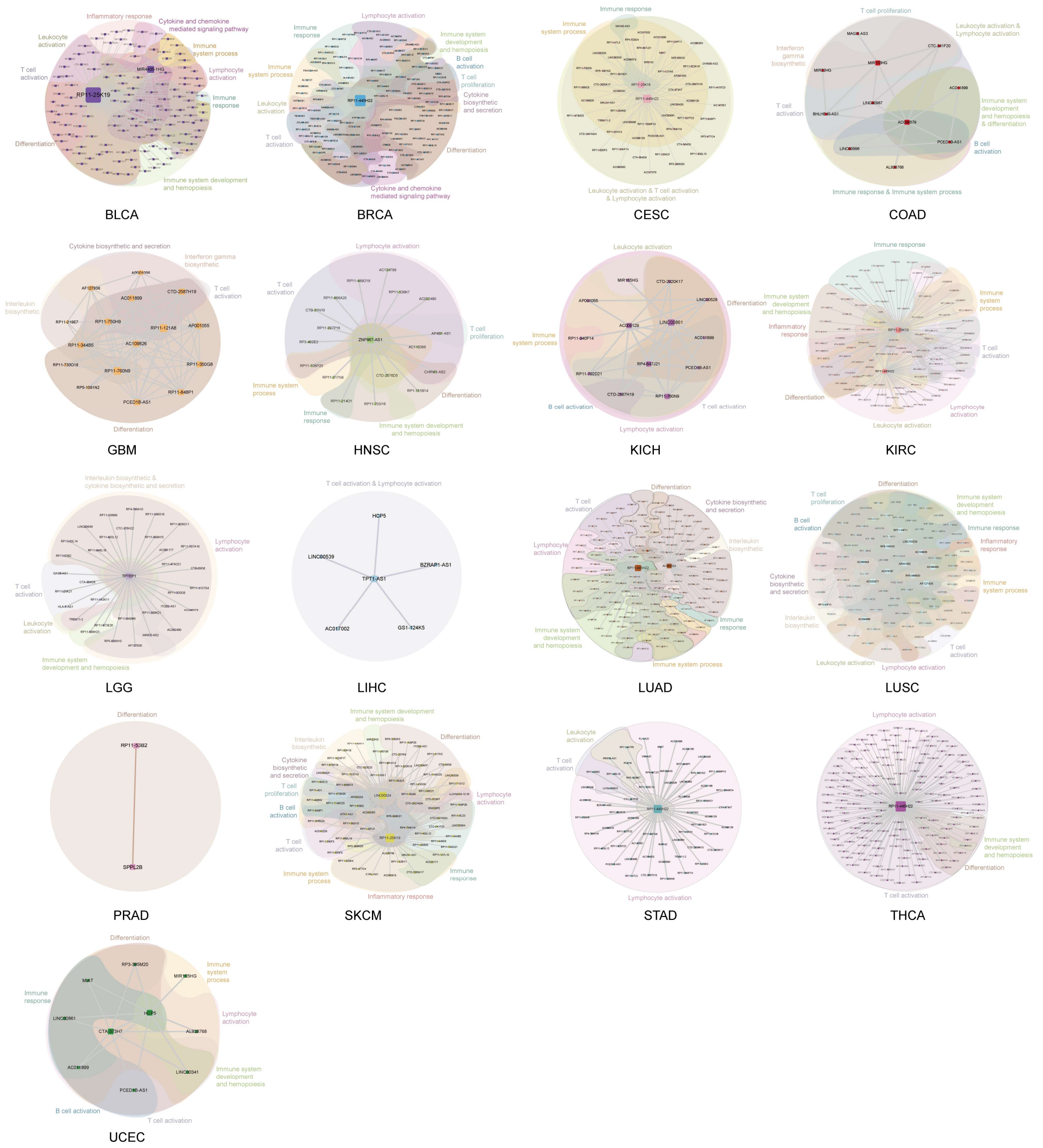

Supplementary Figure 6. LICNs of 17 cancer types. A node represents an IC-lncRNA and an edge represents a cooperative interaction between IC-lncRNAs. A node under the same color shadow shows an IC-lncRNA co-regulating functions belonging to the same function category.

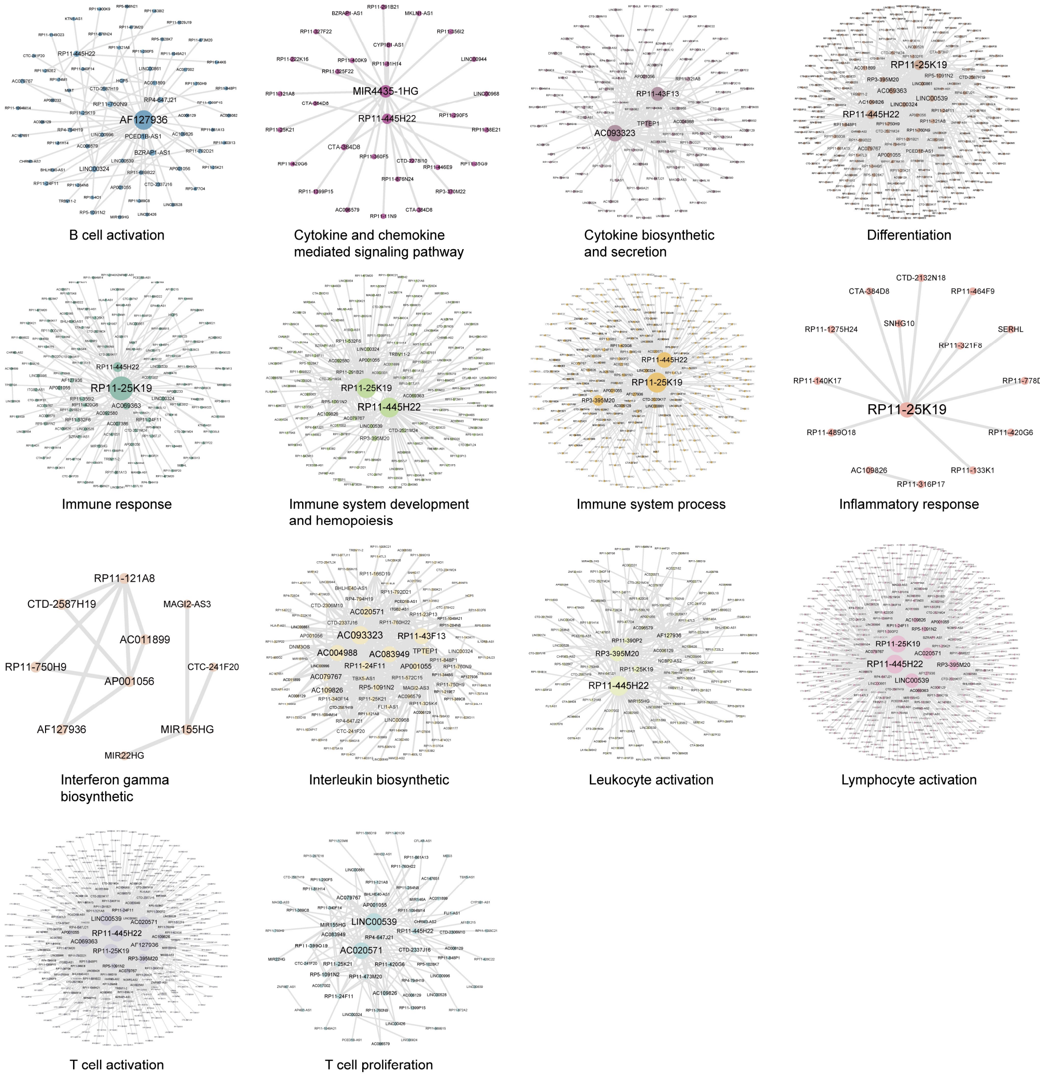

Supplementary Figure 7. LICNs co-regulating 14 immune functions. A node represents an IC-lncRNA and an edge represents a cooperative interaction between IC-lncRNAs.

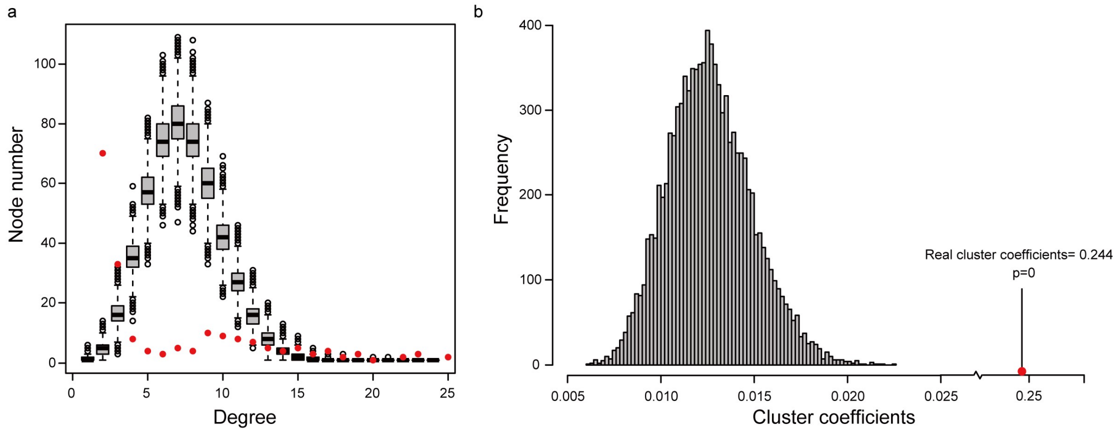

Supplementary Figure 8. The topological characters of LICN. (a) Distribution of degrees for the observed pan-cancer LICN (red circles) and permuted networks (box plots). **(b)** The cluster coefficient of the pan-cancer LICN is higher than those of the random networks.

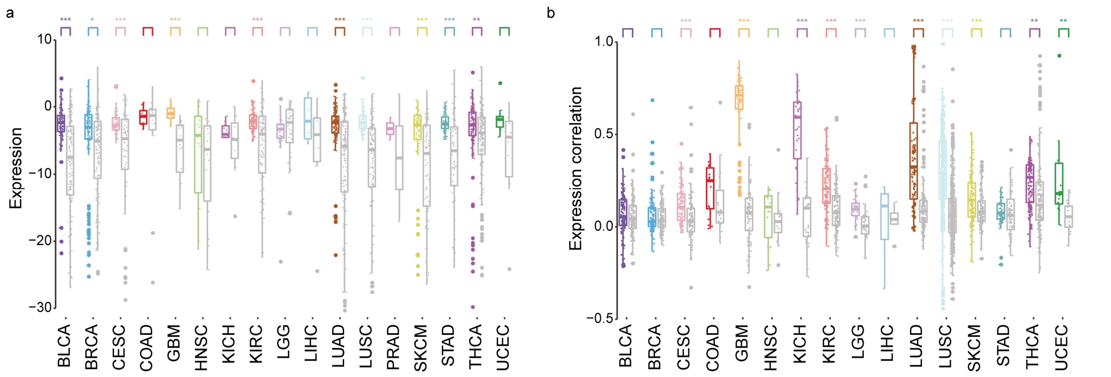

Supplementary Figure 9. The expression features of IC-lncRNAs across cancer types. (a) The differences between expression of IC-lncRNAs and randomly selected lncRNAs. **(b)** The differences of expression correlation between IC-lncRNAs and randomly selected lncRNAs. The gray boxes in a and b represent randomly selected lncRNAs. Two-sided t test was used (* $p < 0.05$, ** $p < 0.01$, *** $p < 0.001$). The n number of IC-lncRNAs per cancer is provided in Supplementary Table 1. The boxplots are shown as median (line), interquartile range (box) and data range or 1.5x interquartile range (whisker), each point indicates a lncRNA.

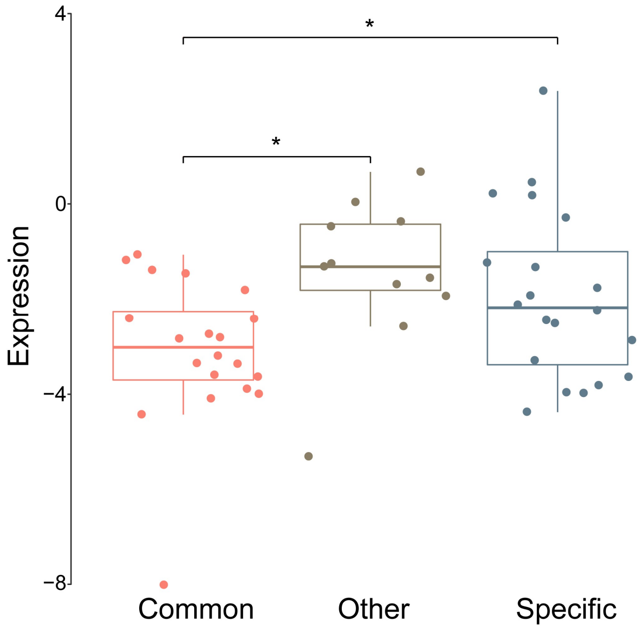

Supplementary Figure 10. Expression of three kinds of hub IC-lncRNAs across cancer types. Two-sided t test was used on n = 20 common hubs, n = 11 other hubs, n = 20 specific hubs, * p<0.05. The boxplots are shown as median (line), interquartile range (box) and data range or 1.5x interquartile range (whisker), each point indicates a hub IC-lncRNA.

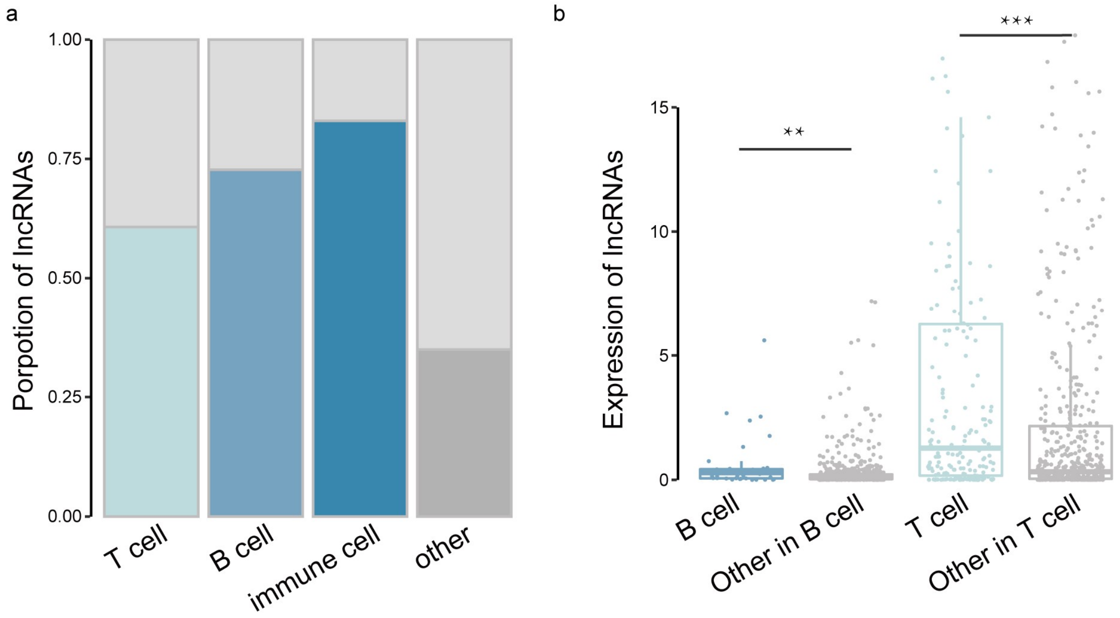

Supplementary Figure 11. The expression of immune lncRNAs in immune cells of scRNA-seq data. (a) The proportion of immune lncRNAs and other lncRNAs expressed in immune cells and immune cell-related lncRNAs expressed in corresponding immune cells of breast cancer in scRNA-seq data (GSE75688, n =515 cells). The lncRNAs were considered as expressed with nonzero count in at least one cells. **(b)** The expression of IC-lncRNAs (n = 40 B cell-related lncRNAs, n = 195 T cell-related lncRNAs) in corresponding immune cells and

other lncRNAs in immune cells in scRNA-seq data of breast cancer (GSE75688). The lncRNAs with nonzero count in at least one cells were obtained. The test was calculated by two-sided Wilcoxon-Mann-Whitney test. ** $p < 0.01$, *** $p < 0.001$. The boxplots are shown as median (line), interquartile range (box) and data range or 1.5x interquartile range (whisker), each point indicates a lncRNA.

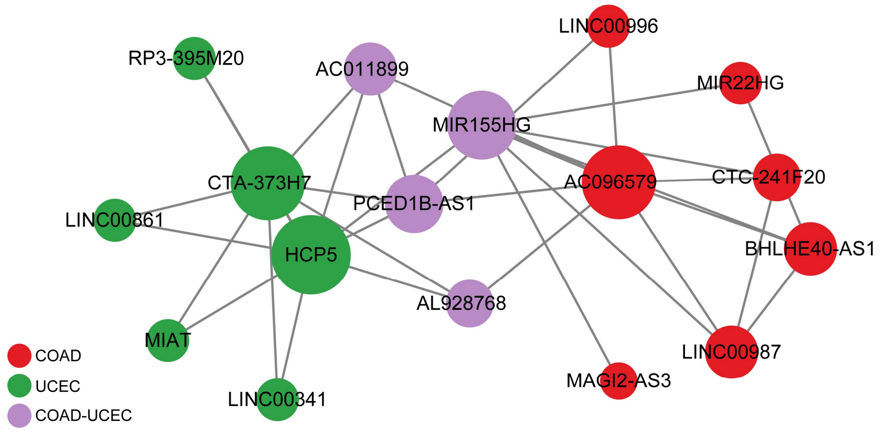

Supplementary Figure 12. The network integrated by LICNs of COAD and UCEC. Nodes in this network can be classified into three categories: IC-lncRNAs only occurred in COAD or UCEC, IC-lncRNAs shared by these two cancer types. An edge represents a cooperative interaction between IC-lncRNAs. All interactions are cancer-context specific.

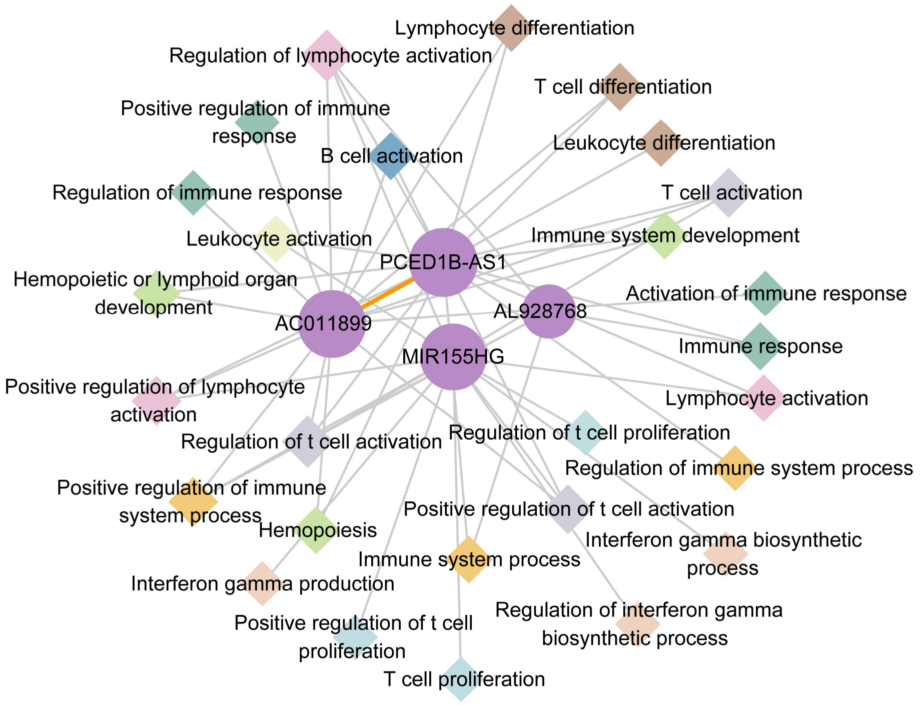

Supplementary Figure 13. The subnetwork consisting of IC-lncRNAs shared by COAD and UCEC and co-regulating functions. A circle shows an IC-lncRNA shared by these two cancer types. A diamond shows an immune function co-regulated by the IC-lncRNAs in each cancer type. The diamond in the same color shows immune functions belonging to the same function category. An edge in orange represents a cooperative interaction between IC-lncRNAs, an edge in gray represents a cooperative interaction between IC-lncRNAs and immune functions.

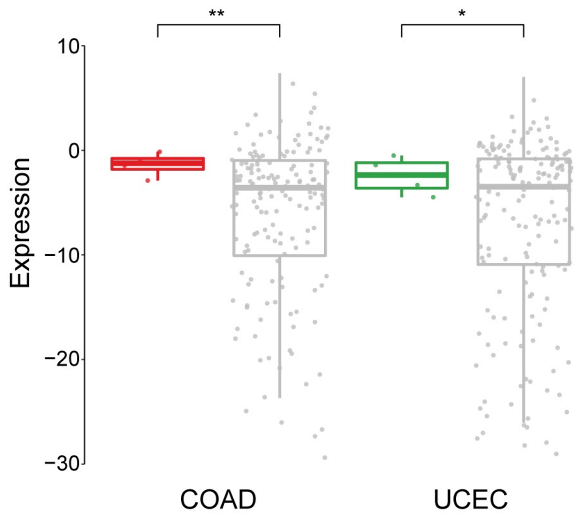

Supplementary Figure 14. The expression of IC-lncRNAs shared by COAD and UCEC in each cancer type. The gray box represents the expression of other lncRNAs in each cancer type. Two-sided t test was used (n = 1577 lncRNAs in COAD, n = 1810 lncRNAs in UCEC). ** p<0.01, * p<0.05. The boxplots are shown as median (line), interquartile range (box) and data range or 1.5x interquartile range (whisker), each point indicates a lncRNA.

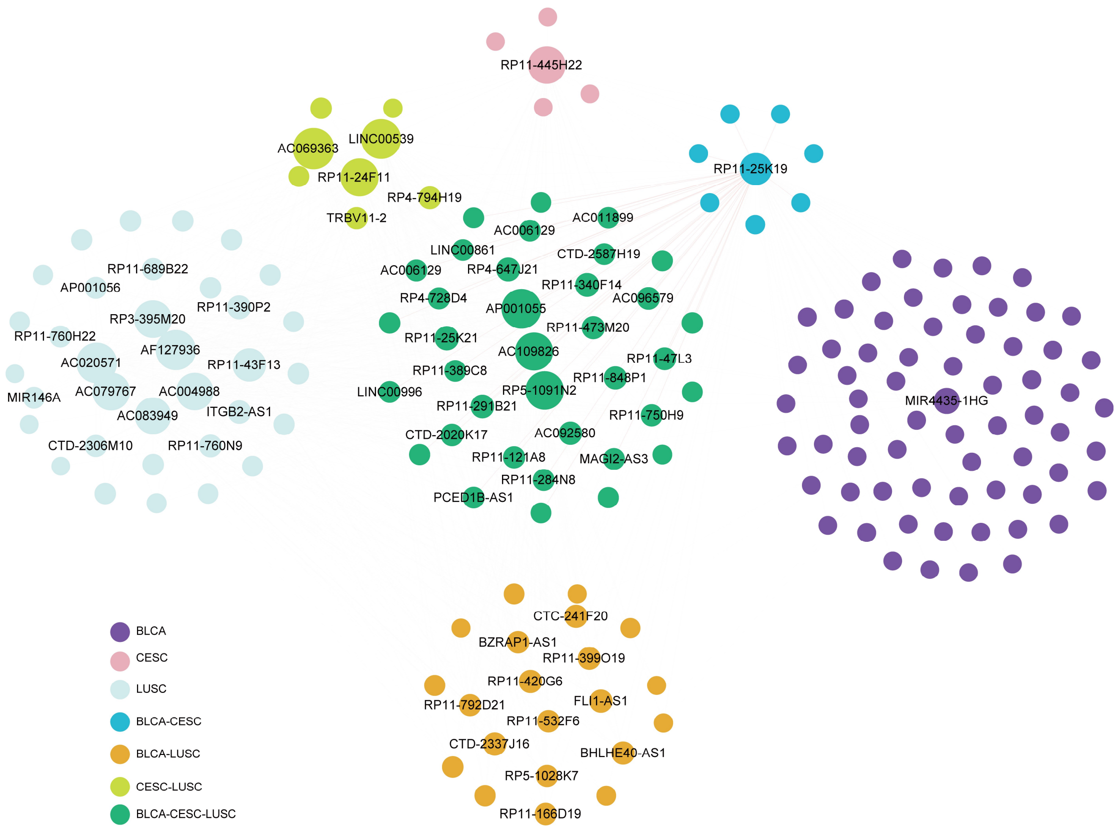

Supplementary Figure 15. The network integrated by LICNs of BLCA, CESC and LUSC. Nodes in this network can be classified into 7 categories: IC-lncRNAs only occurred in BLCA,

CESC or LUSC respectively, IC-lncRNAs shared by any two cancer types, IC-lncRNAs shared by all three cancer types. An edge represents a cooperative interaction between IC-lncRNAs. Edges can be classified into 2 categories: edges in gray color represent interactions only occurred in one cancer type, edges in red color represent interactions shared by BLCA and CESC.

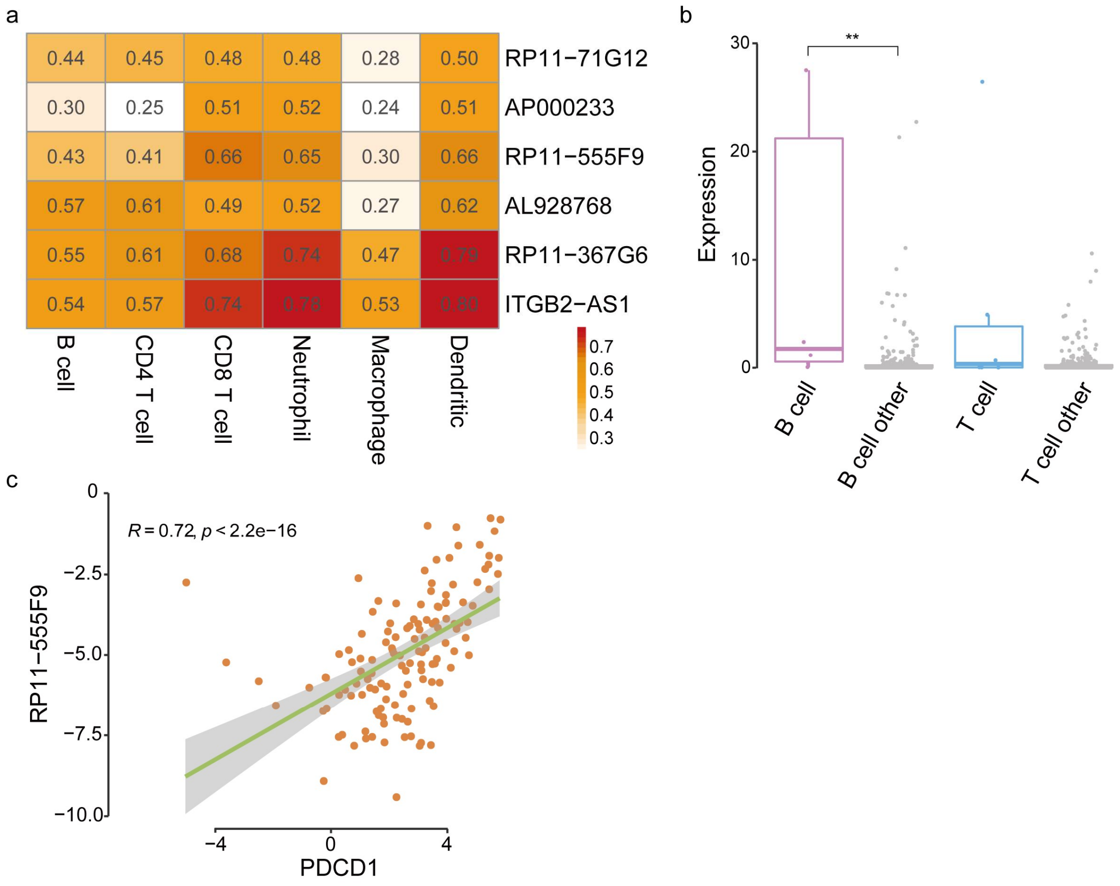

Supplementary Figure 16. Immune subtypes in Skin Cutaneous Melanoma. (a) Spearman's rank correlations between the expression of IC-lncRNAs used to subtype in SKCM and immune cells infiltration. **(b)** Comparison of the expression of signature IC-lncRNAs with other IC-lncRNAs in GEO RNA seq datasets (n = 23 samples of B cell, n = 56 samples of T cell). Two-sided Wilcoxon-Mann-Whitney test was used, ** $p<0.01$. The boxplots are shown as median (line), interquartile range (box) and data range or 1.5x interquartile range (whisker), each point indicates a lncRNA. **(c)** The Pearson correlation of expression between the IC-lncRNA RP11-555F9 and PDCD1.

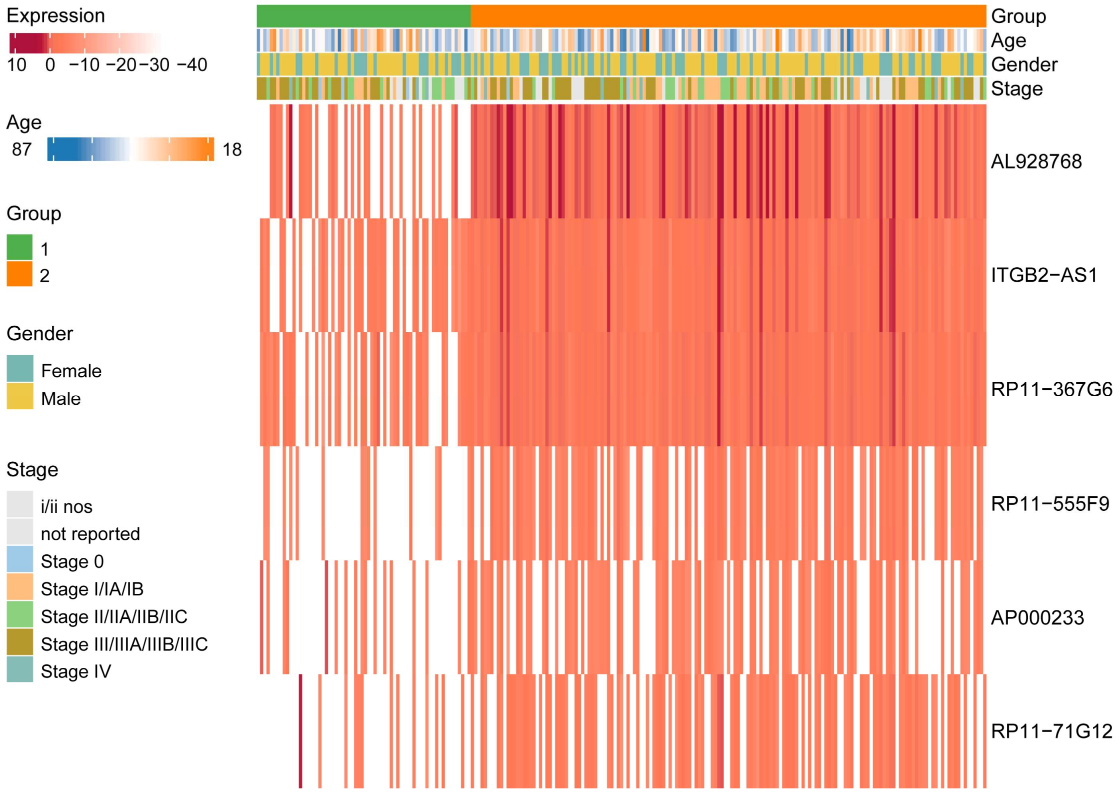

Supplementary Figure 17. The clinical features of patients and the expression of IC-lncRNAs in two subtypes.

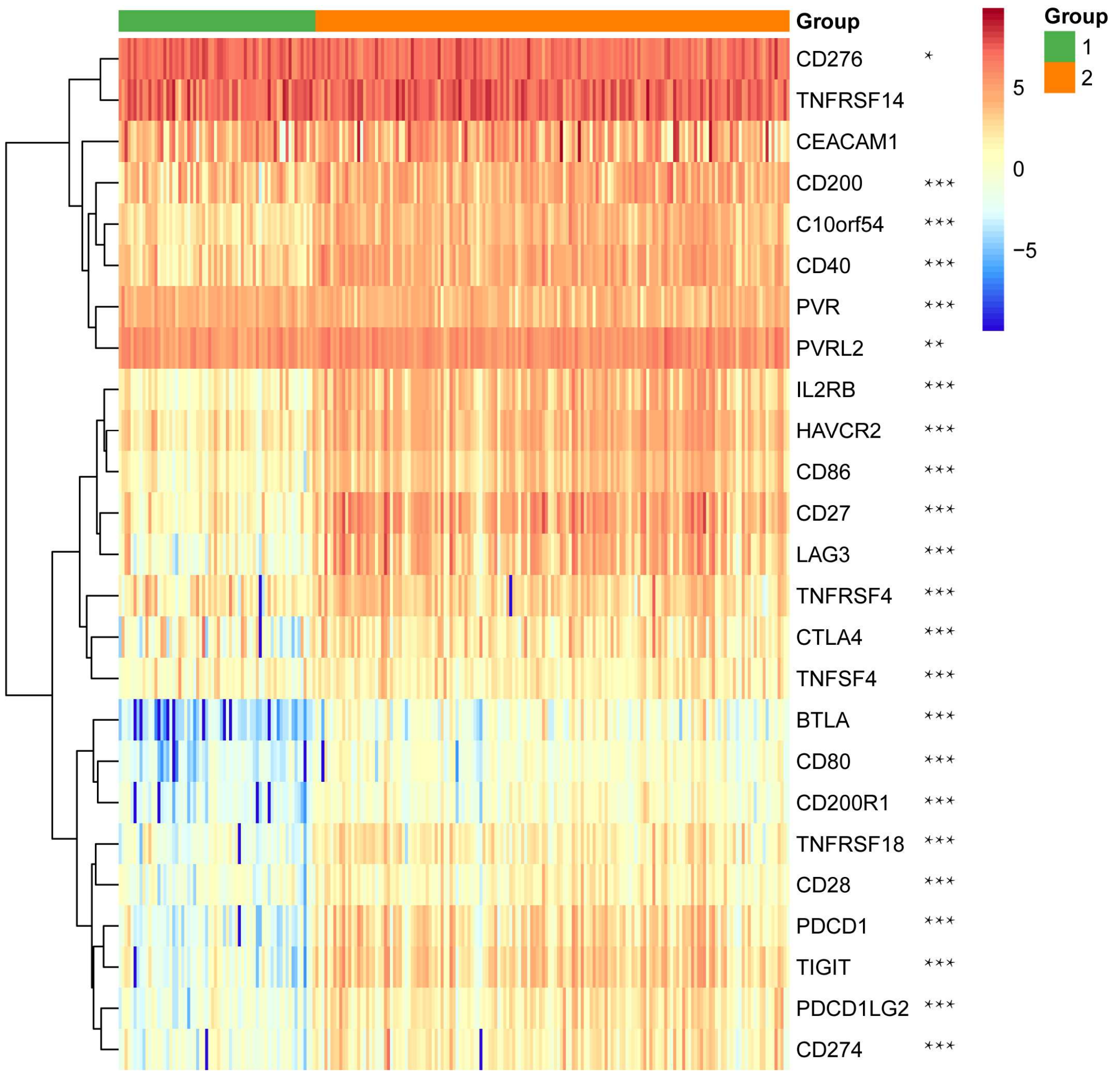

Supplementary Figure 18. The expression of immune checkpoint genes in two subtypes of SKCM. The difference of expression between two subtypes was calculated by t test (group1 = 66 samples, group2 = 159 samples). * p < 0.05, ** p < 0.01, *** p < 0.001.

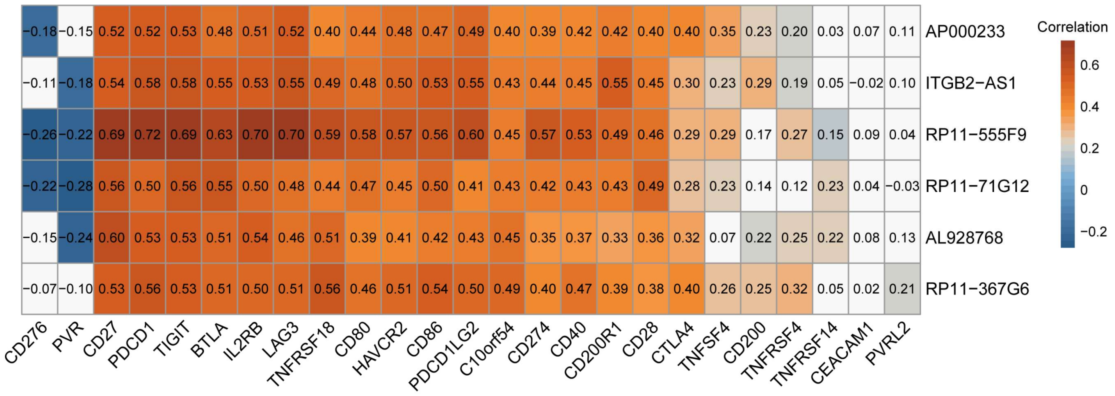

Supplementary Figure 19. The Pearson correlation of the expression between six immune lncRNAs and checkpoint genes in SKCM. The number on colorful background indicates the lncRNA and the checkpoint gene are significantly correlated with p < 0.05. The

numbers on the plot show Pearson coefficients.

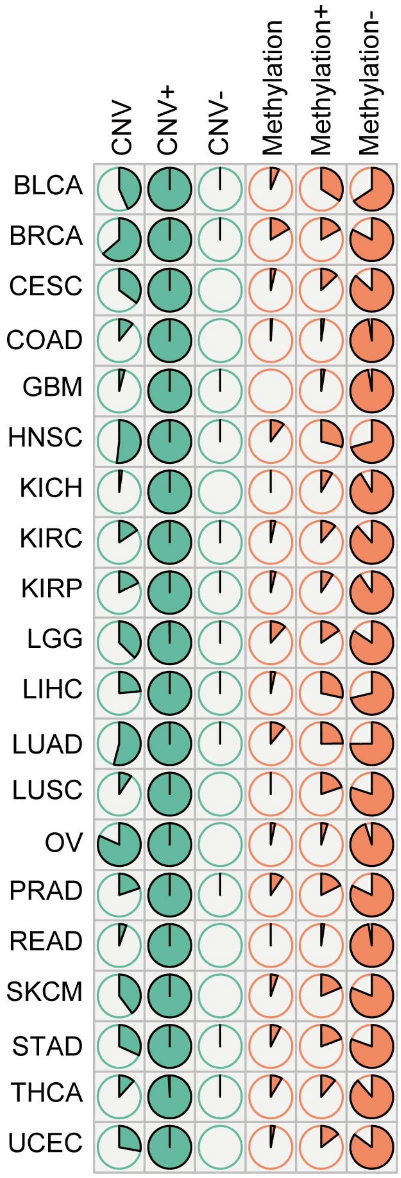

Supplementary Figure 20. The proportion of CNV and promoter methylation in cancers.

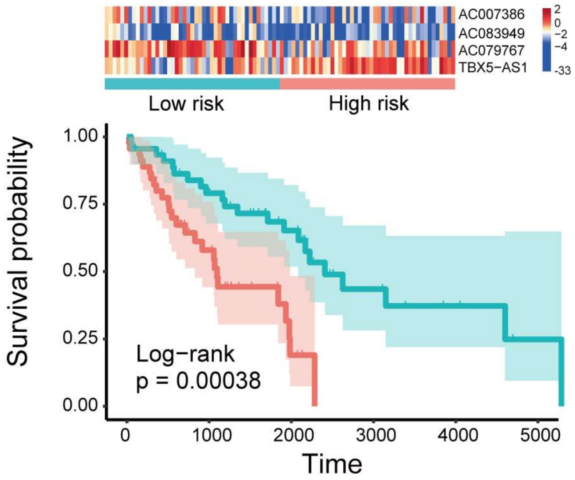

Supplementary Figure 21. The survival analysis of IC-lncRNAs in LUSC. The analysis of survival was performed by the Kaplan-Meier method and difference of survival time was analyzed by the log-rank test (n = 90 samples).

Supplementary Table 1. Summary of analyzed TCGA cancer datasets and cooperative lncRNAs

Cancer	Description	Samples	Co-lncRNA pairs	Co-lncRNAs	IC-lncRNA pairs	IC-lncRNAs
BLCA	Bladder carcinoma	249	249	224	148	130
BRCA	Breast carcinoma	533	350	333	129	130
CESC	Cervical squamous cell carcinoma	191	172	138	91	57
COAD	Colon adenocarcinoma	123	159	81	18	11
GBM	Glioblastoma multiforme	74	963	178	74	16
HNSC	Head and neck squamous cell carcinoma	422	75	79	17	18
KICH	Kidney Chromophobe	66	446	160	31	13
KIRC	Kidney renal clear cell carcinoma	246	471	317	124	101
KIRP	Kidney renal papillary cell carcinoma	181	220	201	0	0
LGG	Lower-grade glioma	484	68	67	33	34
LIHC	Liver hepatocellular carcinoma	196	104	106	5	6
LUAD	Lung adenocarcinoma	428	672	508	147	144
LUSC	Lung squamous cell carcinoma	94	825	151	734	99
OV	Ovarian cancer	407	955	712	0	0
PRAD	Prostate adenocarcinoma	371	46	49	1	2
READ	Rectum adenocarcinoma	65	165	78	0	0
SKCM	Skin cutaneous melanoma	225	375	267	110	73
STAD	Stomach adenocarcinoma	240	155	147	60	61
THCA	Thyroid carcinoma	491	367	355	157	158
UCEC	Uterine Corpus Endometrial Carcinoma	198	212	126	16	10
Total		5284	6449	2145	1628	505

Co-lncRNA: Cooperative lncRNA

Supplementary References

1 Cancer Genome Atlas Research, N. *et al.* The Cancer Genome Atlas Pan-Cancer analysis project. *Nat Genet* **45**, 1113-1120, doi:10.1038/ng.2764 (2013).

2 Li, J. *et al.* TANRIC: An Interactive Open Platform to Explore the Function of lncRNAs in Cancer. *Cancer Res* **75**, 3728-3737, doi:10.1158/0008-5472.CAN-15-0273 (2015).

3 Broad Institute TCGA Genome Data Analysis Center (2015): Firehose stddata__2015_04_02 run. Broad Institute of MIT and Harvard. doi:10.7908/C15X282W.

4 Mermel, C. H. *et al.* GISTIC2.0 facilitates sensitive and confident localization of the targets of focal somatic copy-number alteration in human cancers. *Genome Biol* **12**, R41, doi:10.1186/gb-2011-12-4-r41 (2011).

5 Edgar, R., Domrachev, M. & Lash, A. E. Gene Expression Omnibus: NCBI gene expression and hybridization array data repository. *Nucleic Acids Res* **30**, 207-210, doi:10.1093/nar/30.1.207 (2002).

6 Barrett, T. *et al.* NCBI GEO: archive for functional genomics data sets--update. *Nucleic Acids Res* **41**, D991-995, doi:10.1093/nar/gks1193 (2013).

7 Nordlund, J. *et al.* Genome-wide signatures of differential DNA methylation in pediatric acute lymphoblastic leukemia. *Genome Biol* **14**, r105, doi:10.1186/gb-2013-14-9-r105 (2013).

8 Nordlund, J. *et al.* Digital gene expression profiling of primary acute lymphoblastic leukemia cells. *Leukemia* **26**, 1218-1227, doi:10.1038/leu.2011.358 (2012).

9 Zawada, A. M. *et al.* SuperSAGE evidence for CD14++CD16+ monocytes as a third monocyte subset. *Blood* **118**, e50-61, doi:10.1182/blood-2011-01-326827 (2011).

10 Salzman, J., Gawad, C., Wang, P. L., Lacayo, N. & Brown, P. O. Circular RNAs are the predominant transcript isoform from hundreds of human genes in diverse cell types. *PLoS One* **7**, e30733, doi:10.1371/journal.pone.0030733 (2012).

11 Quintin, J. *et al.* Candida albicans infection affords protection against reinfection via functional reprogramming of monocytes. *Cell Host Microbe* **12**, 223-232, doi:10.1016/j.chom.2012.06.006 (2012).

12 Beyer, M. *et al.* High-resolution transcriptome of human macrophages. *PLoS One* **7**, e45466, doi:10.1371/journal.pone.0045466 (2012).

13 Schmidt, S. V. *et al.* The transcriptional regulator network of human inflammatory macrophages is defined by open chromatin. *Cell Res* **26**, 151-170, doi:10.1038/cr.2016.1 (2016).

14 Pena, O. M. *et al.* Synthetic cationic peptide IDR-1018 modulates human macrophage differentiation. *PLoS One* **8**, e52449, doi:10.1371/journal.pone.0052449 (2013).

15 Wright, H. L., Thomas, H. B., Moots, R. J. & Edwards, S. W. RNA-seq reveals activation of both common and cytokine-specific pathways following neutrophil priming. *PLoS One* **8**, e58598, doi:10.1371/journal.pone.0058598 (2013).

16 Chiewchengchol, D. *et al.* Differential changes in gene expression in human neutrophils following TNF-alpha stimulation: Up-regulation of anti-apoptotic proteins and down-regulation of proteins involved in death receptor signaling. *Immun Inflamm Dis* **4**, 35-44, doi:10.1002/iid3.90 (2016).

17 Qian, F. *et al.* Identification of genes critical for resistance to infection by West Nile virus using RNA-Seq analysis. *Viruses* **5**, 1664-1681, doi:10.3390/v5071664 (2013).

18 Henn, A. D. *et al.* High-resolution temporal response patterns to influenza vaccine reveal a distinct human plasma cell gene signature. *Sci Rep* **3**, 2327, doi:10.1038/srep02327 (2013).

19 Beguelin, W. *et al.* EZH2 is required for germinal center formation and somatic EZH2 mutations promote lymphoid transformation. *Cancer Cell* **23**, 677-692, doi:10.1016/j.ccr.2013.04.011 (2013).
20 Verma, A. *et al.* Transcriptome sequencing reveals thousands of novel long non-coding RNAs in B cell lymphoma. *Genome Med* **7**, 110, doi:10.1186/s13073-015-0230-7 (2015).
21 Seumois, G. *et al.* Epigenomic analysis of primary human T cells reveals enhancers associated with TH2 memory cell differentiation and asthma susceptibility. *Nat Immunol* **15**, 777-788, doi:10.1038/ni.2937 (2014).
22 Zhang, H. *et al.* Functional analysis and transcriptomic profiling of iPSC-derived macrophages and their application in modeling Mendelian disease. *Circ Res* **117**, 17-28, doi:10.1161/CIRCRESAHA.117.305860 (2015).
23 Arlehamn, C. L. *et al.* Transcriptional profile of tuberculosis antigen-specific T cells reveals novel multifunctional features. *J Immunol* **193**, 2931-2940, doi:10.4049/jimmunol.1401151 (2014).
24 Lewis, N. D. *et al.* A GPBAR1 (TGR5) small molecule agonist shows specific inhibitory effects on myeloid cell activation in vitro and reduces experimental autoimmune encephalitis (EAE) in vivo. *PLoS One* **9**, e100883, doi:10.1371/journal.pone.0100883 (2014).
25 Weinstein, J. S. *et al.* Global transcriptome analysis and enhancer landscape of human primary T follicular helper and T effector lymphocytes. *Blood* **124**, 3719-3729, doi:10.1182/blood-2014-06-582700 (2014).
26 Komori, H. K., Hart, T., LaMere, S. A., Chew, P. V. & Salomon, D. R. Defining CD4 T cell memory by the epigenetic landscape of CpG DNA methylation. *J Immunol* **194**, 1565-1579, doi:10.4049/jimmunol.1401162 (2015).
27 Vahedi, G. *et al.* Super-enhancers delineate disease-associated regulatory nodes in T cells. *Nature* **520**, 558-562, doi:10.1038/nature14154 (2015).
28 Pacis, A. *et al.* Bacterial infection remodels the DNA methylation landscape of human dendritic cells. *Genome Res* **25**, 1801-1811, doi:10.1101/gr.192005.115 (2015).
29 Hoek, K. L. *et al.* A cell-based systems biology assessment of human blood to monitor immune responses after influenza vaccination. *PLoS One* **10**, e0118528, doi:10.1371/journal.pone.0118528 (2015).
30 Mitra, S. *et al.* Interleukin-2 activity can be fine tuned with engineered receptor signaling clamps. *Immunity* **42**, 826-838, doi:10.1016/j.immuni.2015.04.018 (2015).
31 Kushwaha, G. *et al.* Hypomethylation coordinates antagonistically with hypermethylation in cancer development: a case study of leukemia. *Hum Genomics* **10 Suppl 2**, 18, doi:10.1186/s40246-016-0071-5 (2016).
32 Litjens, N. H. *et al.* Allogeneic Mature Human Dendritic Cells Generate Superior Alloreactive Regulatory T Cells in the Presence of IL-15. *J Immunol* **194**, 5282-5293, doi:10.4049/jimmunol.1402827 (2015).
33 Cao, Y. *et al.* Functional inflammatory profiles distinguish myelin-reactive T cells from patients with multiple sclerosis. *Sci Transl Med* **7**, 287ra274, doi:10.1126/scitranslmed.aaa8038 (2015).
34 Jiang, K. *et al.* Disease-Associated Single-Nucleotide Polymorphisms From Noncoding Regions in Juvenile Idiopathic Arthritis Are Located Within or Adjacent to Functional Genomic Elements of Human Neutrophils and CD4+ T Cells. *Arthritis Rheumatol* **67**, 1966-1977, doi:10.1002/art.39135 (2015).
35 Zhang, W. C. *et al.* High salt primes a specific activation state of macrophages, M(Na). *Cell Res*

25, 893-910, doi:10.1038/cr.2015.87 (2015).
36 Durrans, A. *et al.* Identification of Reprogrammed Myeloid Cell Transcriptomes in NSCLC. *PLoS One* **10**, e0129123, doi:10.1371/journal.pone.0129123 (2015).
37 Hung, T. *et al.* The Ro60 autoantigen binds endogenous retroelements and regulates inflammatory gene expression. *Science* **350**, 455-459, doi:10.1126/science.aac7442 (2015).
38 Chung, W. *et al.* Single-cell RNA-seq enables comprehensive tumour and immune cell profiling in primary breast cancer. *Nat Commun* **8**, 15081, doi:10.1038/ncomms15081 (2017).
39 Shibru, B. *et al.* Detection of Immune Checkpoint Receptors - A Current Challenge in Clinical Flow Cytometry. *Front Immunol* **12**, 694055, doi:10.3389/fimmu.2021.694055 (2021).
40 Liberzon, A. *et al.* Molecular signatures database (MSigDB) 3.0. *Bioinformatics* **27**, 1739-1740, doi:10.1093/bioinformatics/btr260 (2011).
41 Plaisier, C. L., Pan, M. & Baliga, N. S. A miRNA-regulatory network explains how dysregulated miRNAs perturb oncogenic processes across diverse cancers. *Genome Res* **22**, 2302-2314, doi:10.1101/gr.133991.111 (2012).
42 Keshava Prasad, T. S. *et al.* Human Protein Reference Database--2009 update. *Nucleic Acids Res* **37**, D767-772, doi:10.1093/nar/gkn892 (2009).
43 Wilkerson, M. D. & Hayes, D. N. ConsensusClusterPlus: a class discovery tool with confidence assessments and item tracking. *Bioinformatics* **26**, 1572-1573, doi:10.1093/bioinformatics/btq170 (2010).
44 Gao, Y. *et al.* Lnc2Cancer 3.0: an updated resource for experimentally supported lncRNA/circRNA cancer associations and web tools based on RNA-seq and scRNA-seq data. *Nucleic Acids Res* **49**, D1251-D1258, doi:10.1093/nar/gkaa1006 (2021).
45 Law, V. *et al.* DrugBank 4.0: shedding new light on drug metabolism. *Nucleic Acids Res* **42**, D1091-1097, doi:10.1093/nar/gkt1068 (2014).
