# Supplementary material for: Surveying lncRNA-lncRNA cooperations reveals dominant effect on tumor immunity cross cancers
Source: Commun Biol. 2022 Dec 3;5:1324. doi: 10.1038/s42003-022-04249-0 (PMC9719535; doi:10.1038/s42003-022-04249-0)
Supplement: Supplementary file 2 — Description of Additional Supplementary Data [file 42003_2022_4249_MOESM2_ESM.docx]

**Description of Additional Supplementary Files**

**File name:** Supplementary Data 1

**Description:** lncRNAs that exhibit cooperation in at least 6 cancers.

**File name:** Supplementary Data 2

**Description:** 51 hub IC-lncRNAs.

**File name:** Supplementary Data 3

**Description:** IC-lncRNAs regulating the interactions.

**File name:** Supplementary Data 4

**Description:** Source data used to generate the main figures.
