# Supplementary material for: Surveying lncRNA-lncRNA cooperations reveals dominant effect on tumor immunity cross cancers
Source: Commun Biol. 2022 Dec 3;5:1324. doi: 10.1038/s42003-022-04249-0 (PMC9719535; doi:10.1038/s42003-022-04249-0)
Supplement: Supplementary file 7 — Reporting Summary [file 42003_2022_4249_MOESM7_ESM.pdf]

nature portfolio

Corresponding author(s): Xia Li

Last updated by author(s): Nov 1, 2022

Reporting Summary

Nature Portfolio wishes to improve the reproducibility of the work that we publish. This form provides structure for consistency and transparency in reporting. For further information on Nature Portfolio policies, see our Editorial Policies and the Editorial Policy Checklist.

Statistics

For all statistical analyses, confirm that the following items are present in the figure legend, table legend, main text, or Methods section.

n/a	Confirmed	
☐	☒	The exact sample size (*n*) for each experimental group/condition, given as a discrete number and unit of measurement
☒	☐	A statement on whether measurements were taken from distinct samples or whether the same sample was measured repeatedly
☐	☒	The statistical test(s) used AND whether they are one- or two-sided<br>*Only common tests should be described solely by name; describe more complex techniques in the Methods section.*
☒	☐	A description of all covariates tested
☐	☒	A description of any assumptions or corrections, such as tests of normality and adjustment for multiple comparisons
☐	☒	A full description of the statistical parameters including central tendency (e.g. means) or other basic estimates (e.g. regression coefficient) AND variation (e.g. standard deviation) or associated estimates of uncertainty (e.g. confidence intervals)
☐	☒	For null hypothesis testing, the test statistic (e.g. *F*, *t*, *r*) with confidence intervals, effect sizes, degrees of freedom and *P* value noted<br>*Give P values as exact values whenever suitable.*
☒	☐	For Bayesian analysis, information on the choice of priors and Markov chain Monte Carlo settings
☐	☒	For hierarchical and complex designs, identification of the appropriate level for tests and full reporting of outcomes
☐	☒	Estimates of effect sizes (e.g. Cohen's *d*, Pearson's *r*), indicating how they were calculated

Our web collection on statistics for biologists contains articles on many of the points above.

Software and code

Policy information about availability of computer code

Data collection	The paired lncRNA-mRNA expression profiles as well as DNA methylation and copy number data were obtained from the TCGA Data Portal. The RNA-seq datasets of immune cells were downloaded from GEO (https://www.ncbi.nlm.nih.gov/geo/) under accession number GSE26530, GSE30811, GSE33772, GSE34260, GSE36952, GSE40131, GSE40548, GSE40718, GSE45734, GSE45982, GSE53419, GSE55320, GSE55536, GSE56179, GSE57494, GSE58596, GSE59846, GSE60482, GSE64182, GSE64655, GSE64713, GSE66117, GSE66385, GSE66763, GSE66895, GSE68482, GSE68795 and GSE72502. The Biological Process (BP) terms for Gene Ontology (GO) were downloaded from the MSigDB (v5.1) database (https://www.gsea-msigdb.org/gsea/msigdb/index.jsp). Cancer-related lncRNAs were obtained from lnc2cancer 3.0 (http://bio-bigdata.hrbmu.edu.cn/lnc2cancer/) and literatures (Supplementary Data 2).
Data analysis	The code used in the work is available on GitHub (https://github.com/yunjinxie/stt-lncRNA2022).The statistical significance of differences between groups was evaluated using Wilcoxon-Mann-Whitney test and t test. Fisher's exact test was used to determine whether or not there was a significant association between two categorical variables. The survival difference between groups was assessed by log-rank test. The sample sizes for each cancer were displayed in Supplementary Table 1. A p value<0.05 was considered significant (* p<0.05; ** p<0.01; *** p<0.001; **** p<0.0001). The statistical analyses were performed using RStudio (Version 1.3.1093) with R software version 3.6.3 and 4.0.3.

For manuscripts utilizing custom algorithms or software that are central to the research but not yet described in published literature, software must be made available to editors and reviewers. We strongly encourage code deposition in a community repository (e.g. GitHub). See the Nature Portfolio guidelines for submitting code & software for further information.

Data

Policy information about availability of data

All manuscripts must include a data availability statement. This statement should provide the following information, where applicable:

- Accession codes, unique identifiers, or web links for publicly available datasets
- A description of any restrictions on data availability
- For clinical datasets or third party data, please ensure that the statement adheres to our policy

The paired lncRNA-mRNA expression profiles as well as DNA methylation and copy number data were obtained from the TCGA Data Portal. The RNA-seq datasets of immune cells were downloaded from GEO (https://www.ncbi.nlm.nih.gov/geo/) under accession number GSE26530, GSE30811, GSE33772, GSE34260, GSE36952, GSE40131, GSE40548, GSE40718, GSE45734, GSE45982, GSE53419, GSE55320, GSE55536, GSE56179, GSE57494, GSE58596, GSE59846, GSE60482, GSE64182, GSE64655, GSE64713, GSE66117, GSE66385, GSE66763, GSE66895, GSE68482, GSE68795 and GSE72502. The Biological Process (BP) terms for Gene Ontology (GO) were downloaded from the MSigDB (v5.1) database (https://www.gsea-msigdb.org/gsea/msigdb/index.jsp). Cancer-related lncRNAs were obtained from lnc2cancer 3.0 (http://bio-bigdata.hrbmu.edu.cn/lnc2cancer/) and literatures (Supplementary Data 2). The source data used to generate the main figures is provided in Supplementary Data 4.

Human research participants

Policy information about studies involving human research participants and Sex and Gender in Research.

Reporting on sex and gender	NA
Population characteristics	NA
Recruitment	NA
Ethics oversight	NA

Note that full information on the approval of the study protocol must also be provided in the manuscript.

Field-specific reporting

Please select the one below that is the best fit for your research. If you are not sure, read the appropriate sections before making your selection.

☒ Life sciences ☐ Behavioural & social sciences ☐ Ecological, evolutionary & environmental sciences

For a reference copy of the document with all sections, see nature.com/documents/nr-reporting-summary-flat.pdf

Life sciences study design

All studies must disclose on these points even when the disclosure is negative.

Sample size	No statistical methods were used to predetermine sample size. Patient data we used were acquired by publicly available datasets(TCGA). The sample sizes for each cancer were displayed in Supplementary Table 1.
Data exclusions	We removed mRNAs with a read count less than 20 that were detected in > 95.0% of samples in each cancer type dataset. We removed lncRNA with FPKM less than 0.1 that were detected in > 95.0% of samples in each cancer type dataset.
Replication	Limited dataset are currently available, so no replications have been done.
Randomization	Randomization is not relevant to this work as patient data we used were acquired by publicly available datasets.
Blinding	No blinding was used in this work.

Reporting for specific materials, systems and methods

We require information from authors about some types of materials, experimental systems and methods used in many studies. Here, indicate whether each material, system or method listed is relevant to your study. If you are not sure if a list item applies to your research, read the appropriate section before selecting a response.

Materials & experimental systems

n/a	Involved in the study
☒	☐ Antibodies
☒	☐ Eukaryotic cell lines
☒	☐ Palaeontology and archaeology
☒	☐ Animals and other organisms
☒	☐ Clinical data
☒	☐ Dual use research of concern

Methods

n/a	Involved in the study
☒	☐ ChIP-seq
☒	☐ Flow cytometry
☒	☐ MRI-based neuroimaging
